# Reciprocal projections between the globus pallidus externa and cortex span motor and nonmotor regions

**DOI:** 10.1073/pnas.2423367122

**Published:** 2025-06-03

**Authors:** Emily A. Ferenczi, Wengang Wang, Anushka Biswas, Trent Pottala, Yihuan Dong, Alison K. Chan, Madeline A. Albanese, Raina S. Sohur, Tingying Jia, Kevin J. Mastro, Bernardo L. Sabatini

**Affiliations:** ^a^HHMI, Department of Neurobiology, Harvard Medical School, Boston MA 02115; ^b^Department of Neurology, Massachusetts General Hospital, Harvard Medical School, Boston, MA 02114; ^c^Princeton Neuroscience Institute, Princeton University, Princeton, NJ 08540

**Keywords:** globus pallidus externa, pallidocortical, corticopallidal, reciprocal, nonmotor

## Abstract

The globus pallidus externa (GPe) is a heterogeneous nucleus of the basal ganglia, with intricate connections to other basal ganglia nuclei, as well as direct connections to the cortex. The anatomic, molecular, and electrophysiologic properties of cortex-projecting pallidocortical neurons are not well characterized. Here, we show that pallidocortical neurons project to diverse motor and nonmotor cortical regions, are organized topographically in the GPe, and segregate into at least two distinct electrophysiological and molecular phenotypes. In addition, we find that the GPe receives direct synaptic input from deep layers of diverse motor and nonmotor cortical regions, some of which form reciprocal connections onto pallidocortical neurons. These results demonstrate the existence of a fast, bidirectional circuit between the GPe and the cortex that is ideally positioned to integrate information about behavioral goals, internal states, and environmental cues to rapidly modulate behavior.

## Significance

The existence of direct projections between the globus pallidus externa (GPe) and the cortex places the GPe in a new light as an input/output region of the basal ganglia, rather than an inhibitory relay. We demonstrate that pallidocortical neurons send projections to diverse regions of the cortex, tile the borders of the GPe in a topographic manner, and divide into two distinct electrophysiological and molecular phenotypes. We show that reciprocal projections from the cortex to the GPe arise from deep layers of the motor and nonmotor cortex and can influence GPe neuron activity. These findings inform our understanding of how the cortex modulates, and is modulated by the GPe, and may advance identification of targets for neuromodulatory treatment of basal ganglia disorders.

The globus pallidus externa (GPe) is a central nucleus of the basal ganglia whose function has been implicated in diverse behaviors including regulation of movement and reward-related actions (1–4). The GPe was canonically considered a “motor relay” within the indirect pathway of the basal ganglia, exerting an overall suppressive effect on motor output (5–8). However, it is now apparent that the GPe plays a role in complex goal-directed behavior, mediated by a heterogeneous neural population that forms connections to a broad array of brain regions, both within and outside the basal ganglia (1–3, 9, 10). Understanding how the GPe coordinates and transforms the flow of neural activity within the basal ganglia and to connected brain regions is critical for understanding the pathophysiology of disorders of basal ganglia circuits, such as Parkinson’s disease, dystonia, Tourette’s syndrome, and others (1, 11–21).

In vivo electrophysiological recordings were first performed in the globus pallidus over 60 y ago (22–27). These revealed that activity of pallidal neurons is correlated with features of movement (23–25, 27, 28) and reward (26). Two distinct electrophysiological cellular phenotypes were identified that differ in spontaneous action potential firing rate and bursting properties (23, 25). Since then, the superposition of rich anatomic, molecular, and electrophysiologic information has generated an updated understanding of the complex tapestry of cell types within the GPe (1, 4, 12, 29–37). The current working model subdivides the GPe neural population into two main subtypes. First, the canonical GABAergic “prototypic” neurons, which comprise approximately 70% of GPe neurons, express genetic markers such as *parvalbumin (Pvalb)* and *Nkx2-1* (32, 38), have high spontaneous firing rates, receive inputs from indirect pathway Type 2 dopamine receptor-expressing neurons from the striatum and send inhibitory outputs to traditional striatal indirect pathway output structures such as the substantia nigra pars reticulata (SNr) (4, 23, 29, 30, 32). The second major subtype of GABAergic GPe neurons are referred to as “arkypallidal” neurons. These neurons express *FoxP2*, have lower baseline firing rate, receive input from both the indirect pathway and direct pathway Type 1 dopamine receptor striatal neurons (39) and send output primarily back to the striatum (31, 32, 38–40). Within these two main subclasses, further subdivisions are classified according to the expression of variably overlapping profiles of molecular markers such as *Lhx6* (11, 12, 41), *Npas1* (36, 37, 42, 43), and *Nkx2.1* (36, 38).

Recently, single-cell sequencing of the GPe generated an array of novel genetic markers that segregate the GPe into seven distinct molecular subclusters (44), and detailed anatomic studies have identified rarer GPe neural populations that may be missed with bulk sequencing approaches (33, 36, 45–47). In particular, a small neural population (~5% of GPe neurons) sends a unique direct projection to the cortex and expresses markers for GABAergic and cholinergic neurotransmission (33, 36, 45–49). Recent work has identified additional genetic markers that are expressed by pallidocortical neurons (*Npas1* (36), *Nkx2-1* (36), and *Npr3* (35)) and has shown that pallidocortical neurons predominantly project to frontal regions of the cortex (33, 36, 45). In addition to direct projections from the GPe to the cortex, prior work has demonstrated anatomically (36, 50–53) and confirmed electrophysiologically (36, 50) that the GPe also receives direct synaptic input back from the cortex, including from both the primary and secondary motor cortex (50). The discovery of these pallidocortical circuits has reshaped our understanding of the GPe not as an intermediate relay station within the basal ganglia, but as an output nucleus with the ability to directly modulate cortical activity.

The ability of the GPe to communicate bidirectionally and monosynaptically with the cortex raises critical questions about the role of pallidocortical circuits in integrating information about behavioral goals, internal states, and the external environment to modulate complex behaviors. However, it is important to first understand the detailed properties and organization of pallidocortical circuits. Previous work has shown that topographic heterogeneity of the GPe is layered over its molecular heterogeneity (54, 55); however, our understanding of the topographic organization of pallidocortical neurons, the extent of cortical regions to which they project, and the specificity of reciprocal topographic input from the cortex back to the GPe remains incomplete.

The presence of pallidocortical and corticopallidal circuits is relevant for human disease, as white matter tracts connecting cortical to basal ganglia structures have become important targets for surgical intervention for neurologic and psychiatric disease. In humans, the “motor hyperdirect” pathway from the motor cortex directly to the STN is considered a key target for the therapeutic benefits of deep brain stimulation (DBS) in Parkinson’s disease (56, 57). Other “hyperdirect” white matter bundles coursing from cortical regions such as the cingulate cortex to the basal ganglia have been postulated as DBS targets for other neuropsychiatric conditions such as obsessive compulsive disorder (58). Understanding the detailed molecular, anatomic, and physiological properties of circuits between the GPe and the cortex will enhance our ability to identify and optimize novel approaches and targets for neuromodulatory therapy.

Here, we examined the anatomic and topographic distribution of pallidocortical neurons within the GPe and correlated their electrophysiological properties with their cortical projection target. In addition, we examined the molecular markers that characterize anatomically defined pallidocortical neurons. Finally, we examined whether pallidocortical neurons receive direct monosynaptic input from the cortex, and if so, how this input differs from cortical input to other GPe cell types. Our findings indicate that pallidocortical neurons project to both motor and nonmotor regions of the cortex, and their topography is reflected in their cortical projection target. Pallidocortical neurons segregate into at least two distinct electrophysiological and molecular subtypes and receive direct monosynaptic excitatory input back from motor and nonmotor regions of the cortex, which is comparable in magnitude and latency to cortical input to other GPe neurons.

## Results

### Pallidocortical Neurons Encircle the GPe with a Topography That Depends Upon Cortical Projection Target.

**Fig. 1.**
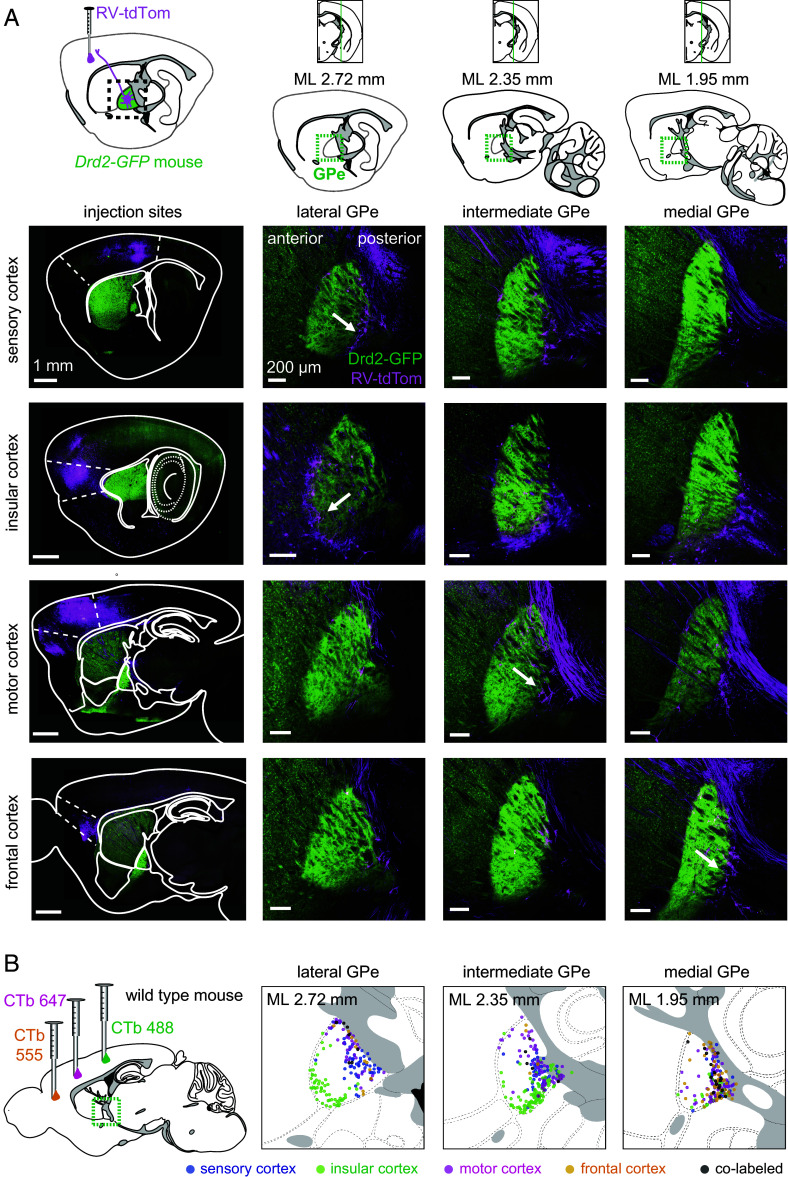
Pallidocortical neurons encircle the GPe. (*A*) *Top,* Schematic of the experimental approach: nonpseudotyped tdTom-expressing rabies virus (RV-tdTom) was injected into one of four cortical regions (sensory cortex, motor cortex, insular cortex, frontal cortex) in *Drd2*-*GFP* mice. *Bottom,* Sagittal section fluorescence images. Injection sites are shown in the *Left* panel. RV-infected cells (magenta) in the GPe (green) are shown at lateral, intermediate, and medial planes through the GPe (arrows provide examples). The GPe is highlighted by GFP fluorescence in indirect pathway (*Drd2*) axons (n = 4 mice, 1 per cortical injection site). (*B*) Cholera-toxin b (CTb) tagged with different fluorophores was injected into three different cortical regions in each mouse. The location of CTb-labeled cells in the GPe annotated on sagittal section diagrams from the Allen Brain Atlas (60) (n = 563 cells across 6 mice, 3 cortical injections per mouse).

Prior studies have suggested that pallidocortical neurons congregate at the medial and ventral borders of the GPe (33, 45–47), but it is unknown whether they maintain the spatial topography of their cortical targets. To determine whether the spatial distribution of pallidocortical neurons varies according to their cortical projection target, we sampled four different cortical regions, including both sensorimotor regions—primary and secondary motor cortex (from now on referred to as “motor cortex”), primary somatosensory cortex (“sensory cortex”)—and higher-order association areas—anterior cingulate cortex, prelimbic and infralimbic cortex (“frontal cortex”) and dorsal agranular insular cortex and gustatory (dysgranular and granular) insular cortex (“insular cortex”). We injected a retrograde fluorophore-encoding, G-deleted, nonpseudotyped rabies virus (RV) into each of these cortical regions to determine the spatial distribution of pallidocortical neuron cell bodies in the GPe (Fig. 1*A*). This virus directly infects axons in the cortex and expresses a fluorophore to fill the cell body and reveal its location. The injections were performed in *Drd2-GFP* transgenic mice, in which the pattern of GFP expression highlights the GPe (59) (*SI Appendix*, Table S1).

We observed that many pallidocortical neurons encircle the GPe in a ring-like manner, particularly around the ventral and posterior borders of the GPe, with insular cortex-projecting neurons aligning predominantly along the ventral and anterolateral borders of the GPe, and sensory, prefrontal, and motor cortex-projecting neurons aligning in partially overlapping clusters along the posteromedial borders (Fig. 1*A*). In addition, cells labeled from all projection targets were found within the core of the GPe. To verify that this spatial segregation was not a confound of viral tropism, we performed a similar experiment using a nonviral retrograde tracer, cholera toxin b (CTb) (Fig. 1*B*). Using CTb labeled with three different fluorophores, we assessed the topography of pallidocortical neurons projecting to three different cortical regions simultaneously and obtained similar results as with RV (Fig. 1*B* and *SI Appendix*, Fig. S1*C*). Only a small proportion of neurons were colabeled with more than one CTb fluorophore, suggesting that GPe neurons tend to project to specific cortical areas, rather than sending wide-reaching collaterals across multiple different cortical regions (*SI Appendix*, Fig. S1 *C* and *D*). Although underlabeling of pallidocortical neurons may underestimate the fraction of cells that innervated multiple regions, a positive control with injection of two different CTb fluorophore labels into the same cortical location generated a high proportion of colabeled pallidocortical neurons (*SI Appendix*, Fig. S2 *A*–*D*).

### Pallidocortical Neurons Fall into Two Distinct Electrophysiological Clusters That Do Not Depend Upon Cortical Projection Target.

**Fig. 2.**
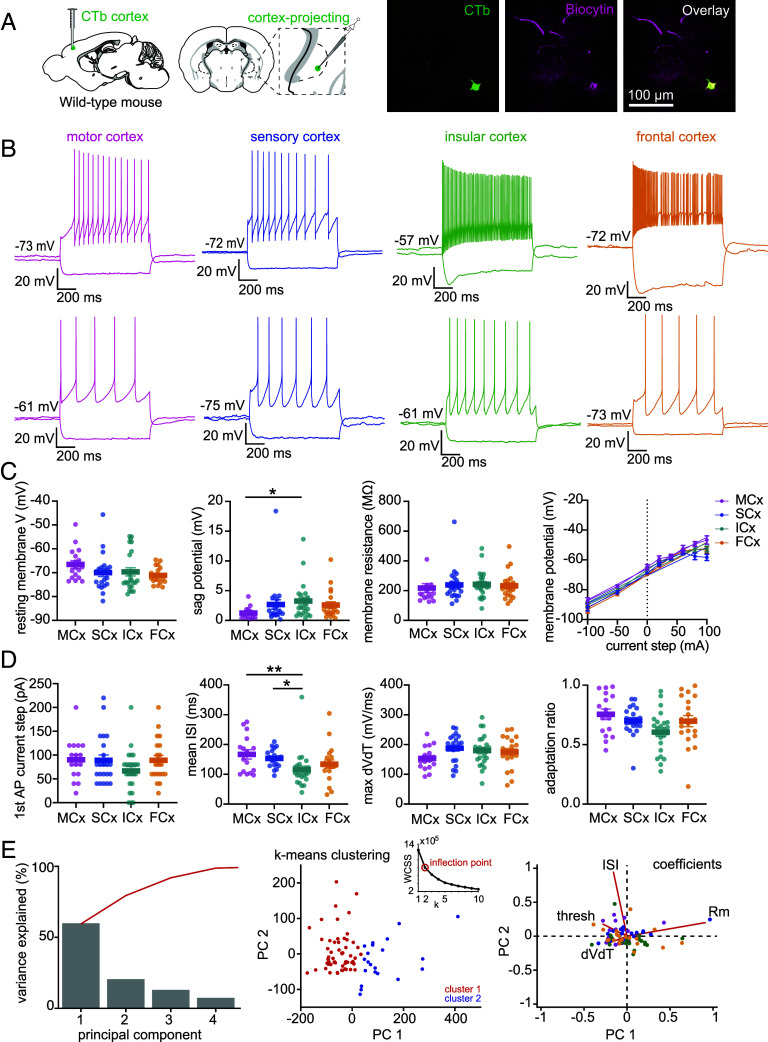
Pallidocortical neurons fall into two distinct electrophysiological clusters. (*A*) *Left,* Schematic of the experimental approach: Retrograde labeling of pallidocortical neurons with CTb from the motor cortex (n = 3 mice), sensory cortex (n = 3 mice), insular cortex (n = 3 mice), and frontal cortex (n = 4 mice). *Right,* Example of a CTb-positive (green) and biocytin-positive (magenta) cell. (*B*) Example current clamp recordings from pallidocortical neurons projecting to each of the four cortical regions. A hyperpolarizing and a suprathreshold depolarizing response is shown for each cell. Example traces were chosen to illustrate the extent of variability of firing rates and sag responses of neurons projecting to the four different cortical regions. (*C*) Quantification of passive properties of pallidocortical neurons according to cortical projection target: resting membrane potential (resting membrane V), sag potential (sag V), membrane resistance (Rm), and current–voltage relationship. Sample size for each region: motor cortex n = 17 cells, 3 mice, sensory cortex n = 22 cells, 3 mice, insular cortex n = 24 cells, 3 mice, frontal cortex n = 20 cells, 4 mice. Mean and SEM are shown. There was a significant difference between groups for sag potential [Kruskal–Wallis (K-W)=9.102, *P* = 0.028, significant for motor cortex vs insular cortex (*P* = 0.0180), after multiple comparison corrections]. (*D*) Quantification of active properties of pallidocortical neurons according to cortical projection target: current step required to generate a first action potential (1st AP current step), mean interspike interval (ISI), mean action potential rate of voltage change (mean max dVdT), mean adaptation ratio (first AP interval/last AP interval). Mean and SEM are shown. There was a significant difference between groups for mean ISI [Kruskal–Wallis (K–W) = 16.65, *P* = 0.0008, *P* = 0.0213, significant for motor cortex vs insular cortex (*P* = 0.0107) and sensory cortex vs insular cortex (*P* = 0.0014), after multiple comparison corrections]. (*E*) Principal component analysis (PCA) of electrophysiological properties (n = 83 cells, 13 mice). *Left,* Scree plot. Percent variance in the data explained by the first four principal components. *Middle,* K-means clustering for k = 2 clusters, data points are color-coded by cluster assignment. *Inset:* Elbow plot of Within-Cluster Sum of Squares (WCSS) for different values of k. The maximum of the ratio of the second derivative/first derivative was used to identify the optimal value for k (the inflection point of the curve). *Right*, Biplot of the first two principal components (PC1 and PC2) showing variable coefficients and scores. Points represent the scores for each cell on PC1 and PC2 and are colored according to cortical projection target. Red lines indicate the direction and magnitude of variable coefficients, the line length is proportional to the contribution of each variable to the principal components. *Abbreviations*, thresh: current step to elicit first action potential, dVdT: action potential waveform acceleration, ISI: interspike interval, Rm: membrane resistance.

The functional impact of synaptic input from the GPe to the cortex (33, 45) will depend in part upon the intrinsic electrophysiological properties of pallidocortical neurons. Prior work has shown that *Chat+* and *Slc32a1+* (vesicular GABA transporter) GPe neurons have distinct electrophysiological phenotypes (45); however, it is unknown whether pallidocortical neurons segregate into distinct electrophysiological subtypes according to their cortical target. To examine this, we retrogradely labeled pallidocortical neurons according to cortical projection target with CTb and performed whole-cell electrophysiological current clamp recordings ex vivo from CTb-labeled neurons in the GPe in acute brain slices (Fig. 2*A*). These recordings revealed that pallidocortical neurons exhibit a wide variability in electrophysiological properties. Some neurons exhibit slow, regular spiking, whereas others showed high frequency and adapting spiking with varying degrees of a sag response to hyperpolarization (Fig. 2 *B*–*D*). The example traces (Fig. 2*B*) highlight the extent of this variability, but many neurons exhibited intermediate properties. This variability in intrinsic properties across neurons did not clearly correspond to differences in cortical projection targets. Nevertheless, some differences exist between motor/sensory cortex-projecting neurons and insular cortex-projecting neurons in terms of their response to hyperpolarization (sag potential is greater for insular cortex-projecting neurons) (Fig. 2*C*), and their interspike intervals (ISIs) during a spike train (shorter for insular cortex-projecting neurons) (Fig. 2*D*). Principal component analysis (PCA) across a wide range of electrophysiological properties (Fig. 2*E* and *SI Appendix*, Fig. S3*C*) revealed that when analyzed independently of cortical projection target, pallidocortical neurons cluster into two adjacent but distinct phenotypes, predominantly separated by action potential firing properties (ISI, current step for first spike, and action potential acceleration), and membrane resistance (Fig. 2*E* and *SI Appendix*, Fig. S3*C*).

### There Are at Least Two Main Molecular Phenotypes of Pallidocortical Neurons.

**Fig. 3.**
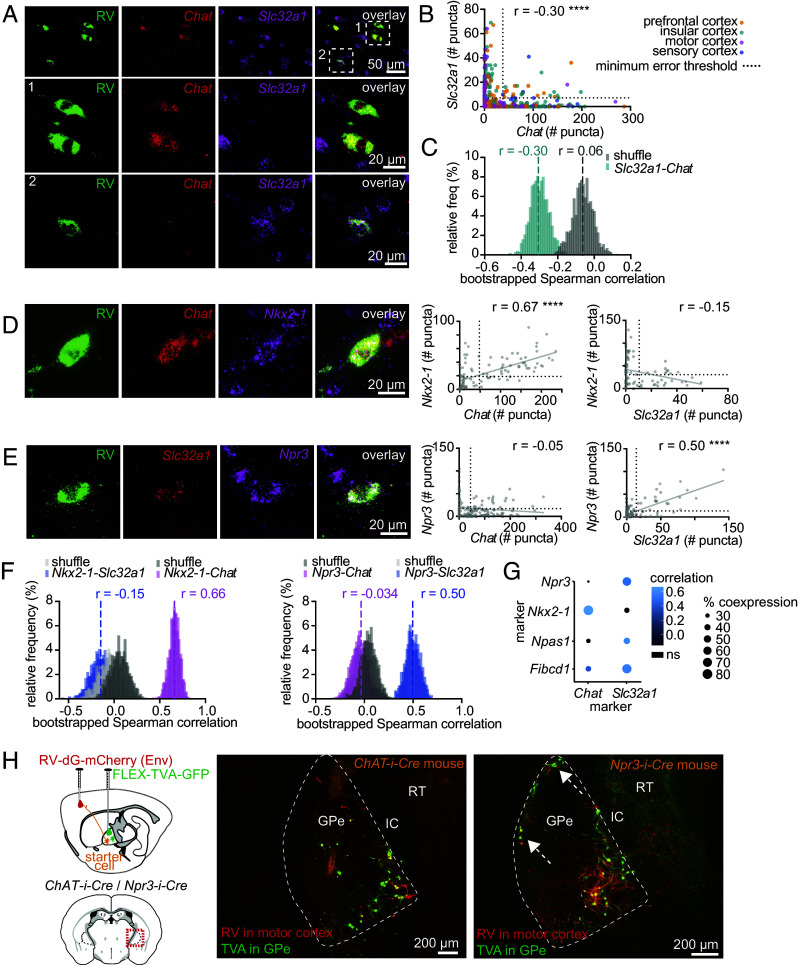
Two molecular classes of pallidocortical neurons. (*A*) Rabies virus (RV) was used to retrogradely label pallidocortical neurons. *Top row,* Examples of mRNA fluorescent in situ hybridization (mRNA-FISH), with labeling of RV *gp1* (*RV*) and markers for cholinergic (*Chat*—choline acetyl transferase) and GABAergic (*Slc32a1*—vesicular GABA transporter) neurotransmission. *Middle row,* Enlarged image of a *Chat*-positive/*Slc32a1*-negative RV-infected neuron. *Bottom row,* Enlarged image of a *Chat*-negative/*Slc32a1*-positive RV-infected neuron. (*B*) Quantification of mRNA puncta numbers for *Chat* and *Slc32a1* in RV-infected pallidocortical neurons. Each dot represents a different neuron, colored according to its cortical projection target. The minimum error threshold classifies neurons into expressing or nonexpressing for each marker. Spearman rho (r) correlation coefficient for number of *Chat* puncta versus *Slc32a1* puncta r = −0.30 (*P* < 0.0001, n = 326 cells, 4 mice) (*C*) Bootstrap analysis for Spearman correlation coefficients for nonshuffled and shuffled data: mean nonshuffled r = −0.30, mean shuffled r = −0.06 (SD: 0.056, number of bootstraps = 1,000). (*D*) Example images of mRNA-FISH with labeling of *RV*, *Chat,* and *Nkx2-1*. Quantification of mRNA puncta for *Nkx2-1* vs *Chat* [Spearman rho r = 0.67 (*P* < 0.0001, n = 106 cells, 1 mouse) and *Nkx2-1* vs *Slc32a1* (Spearman rho r = −0.15, *P* = 0.2, n = 75 cells, 1 mouse)]. (*E*) Example images of mRNA-FISH with labeling of *RV*, *Slc32a1,* and *Npr3*. Quantification of mRNA puncta for *Npr3* vs *Chat* (Spearman rho, r = −0.05 (*P* = 0.51, n = 159 cells, 1 mouse) and *Npr3* vs *Slc32a1* (Spearman rho r = 0.50, *P* < 0.0001, n = 126 cells, 1 mouse). (*F*) Bootstrap analysis for Spearman correlation coefficients for nonshuffled and shuffled data for *Nkx2-1* vs *Chat* and *Nkx2-1* vs *Slc32a1; Npr3* vs *Chat* and *Npr3* vs *Slc32a1.* Mean correlation coefficients for nonshuffled data are shown, along with distribution of shuffled and nonshuffled bootstrapped data. Distributions of nonshuffled data for *Nkx2-1* vs *Chat* and *Npr3* vs *Slc32a1* are separated from their respective shuffled distributions by more than 2 SD. (*G*) Dot plot summary for correlation coefficients and percent coexpression between different GPe markers in pallidocortical neurons. Nonsignificant correlation coefficients are indicated in black. (*H*) Confirmation that *Chat* and *Npr3* can be used to label motor cortex-projecting pallidocortical neurons in the GPe. Experimental approach and example images of *Cre-*dependent TVA-expressing (green) and rabies-expressing pallidocortical neurons (red/yellow) in the GPe in *Chat-i-Cre* (*Left)* and *Npr3-i-Cre mice* (*Right*). Arrows highlight Npr3+ pallidocortical neurons located at the lateral borders of the GPe.

Since intrinsic electrophysiological variation between pallidocortical neurons was not primarily determined by cortical projection target, we hypothesized that these differences result from variation in the molecular identity of pallidocortical neurons. To evaluate whether there are distinct molecular identities of pallidocortical neurons, we performed in situ mRNA hybridization on brain slices containing the GPe, in which pallidocortical neurons were labeled using a retrograde nonpseudotyped RV tracer (Fig. 3). Based on prior literature, we expected pallidocortical neurons to express markers for cholinergic and GABAergic neurotransmission (33, 45) and indeed confirmed that *Slc32a1 and Chat* mRNA colocalized with RV mRNA (*gp1* gene) (Fig. 3 *A*–*C*). 64% of all RV-infected cells expressed at least one marker, and of these, a small proportion (11.5%) coexpressed *Chat* and *Slc32a1* (17% *of* RV-infected *Chat+* neurons expressed *Slc32a1*, and 24% of RV-infected *Slc32a1+* neurons expressed *Chat*).

Since *Slc32a1* is expressed by many GPe neurons, we focused on identifying other genetic markers to specifically delineate this noncholinergic population. We selected known GPe markers for pallidocortical neurons: *Npas1* (36, 42, 43)*, Nkx2-1* (36), as well as novel GPe markers *Npr3* (36, 44) and *Fibcd1* (44, 60) which, based on their sparsity and anatomic distribution, have potential to mark pallidocortical neurons (*SI Appendix*, Fig. S6*C*). We did not investigate other well-characterized GPe markers such as *Pvalb* and *FoxP2*, which are minimally expressed by pallidocortical neurons (36, 45), and *Lhx6,* which is expressed by multiple GPe neuron subtypes (12, 32, 35, 36, 38, 44, 61). We found that *Fibcd1* was expressed by both *Chat*+ and *Slc32a1+* neurons, whereas *Nkx2-1* expression was restricted to *Chat+* neurons, and *Npr3* and *Npas1* expression was restricted to *Slc32a1*+ neurons. Overall, *Npr3* and *Nkx2-1* sharply demarcated pallidocortical neurons into at least two distinct molecular subtypes—*Npr3+/Slc32a1+* neurons, and *Nkx2-1+/Chat+* neurons (Fig. 3 *D*−*G*).

To confirm that our mRNA in situ hybridization experiments reliably identified genetic markers that selectively label pallidocortical neurons, we used pseudotyped RV to retrogradely label pallidocortical neurons in a genetically specified manner (Fig. 3*H* and *SI Appendix*, Fig. S5). Pseudotyped RV contains an envelope protein (EnvA) that requires a complementary receptor (avian tumor virus receptor A, TVA) to infect axonal terminals. Virally mediated TVA expression can be made *Cre*-recombinase dependent to restrict rabies infection to only the subset of neurons that express *Cre*. We injected a *Cre*-dependent TVA helper virus into the GPe of *Npr3-i-Cre* (*Npr3-IRES-Cre*) or *ChAT-i-Cre (ChAT-IRES-Cre)* transgenic mice (*SI Appendix*, Table S1), to drive TVA expression selectively in either Npr3-positive (Npr3+) or ChAT-positive (ChAT+) neurons. Two weeks later, we injected EnvA pseudotyped, G-deleted RV into either motor or insular cortex of the same mice (Fig. 3*H* and *SI Appendix*, Fig. S5 *A*–*C*). This resulted in strong RV labeling in GPe neurons in transgenic mice (Fig. 3*H* and *SI Appendix*, Fig. S5*A*), but only minimal and sparse labeling in wild-type control mice (*SI Appendix*, Fig. S5*F*), confirming the existence of cortically projecting Npr3+ and ChAT+ cells in the GPe. We saw Npr3+ and ChAT+ rabies-labeled neurons in the GPe for both motor cortex and insular cortex RV injections (Fig. 3*H* and *SI Appendix*, Fig. S5*A*), demonstrating that the broad projections to the motor and nonmotor cortex are a property of both molecular subtypes of pallidocortical neuron, and cannot be ascribed to the cholinergic projection alone.

This cell type–specific rabies tracing approach also allowed us to visualize axonal collaterals of Npr3+ and ChAT+ neurons in other brain regions. However, there are two important limitations to consider: 1) the axonal projections are sparse, 2) *Npr3* (and *Chat* to a lesser degree) are expressed in the cortex resulting in some expression of TVA and RV in regions around the cortical injection site, particularly in *Npr3-i-Cre* mice. This could lead to spurious axonal projections arising from cortical neurons rather than GPe neurons. Nevertheless, qualitatively, we observed two prominent subcortical axonal projections and performed additional control experiments to confirm that these originated from the GPe.

First, in *Npr3-i-Cre*, TVA+/RV+ axons were seen in the reticular nucleus of the thalamus (RT), consistent with prior reports (35, 36). Labeled axons in RT were present in mice injected with RV in both the motor cortex and insular cortex, suggesting that *Npr3+* pallidocortical neurons project to RT, regardless of their cortical target. In contrast, ChAT+ axons were not seen in the RT for either projection target (*SI Appendix*, Fig. S5 *B* and *C*). To confirm that TVA/RV-labeling in RT originated from GPe neurons rather than cortical neurons, we injected *Cre*-dependent TVA-GFP helper virus only into the cortex of a *Npr3-i-Cre* mouse (no GPe injection) and detected a TVA-GFP+ axonal projection coursing through the internal capsule, but no TVA-labeled fibers in the RT (*SI Appendix*, Fig. S5*E*). Second, in *Npr3-i-Cre* and *ChAT-i-Cre* mice injected with *Cre*-dependent TVA-GFP helper virus in the GPe and pseudotyped (EnvA) RV in the insular cortex, RV-mCherry labeled axons were found in the amygdala, but not in mice injected with RV in the motor cortex. To confirm that these projections to the amygdala originated from GPe neurons and not directly from neurons in the insular cortex, we injected *Cre*-dependent TVA-GFP helper virus in the GPe in *ChAT-i-Cre* and *Npr3-i-Cre* mice followed by RV injection into the amygdala to assess for cell body labeling in the GPe (*SI Appendix*, Fig. S5*D*). We observed RV-labeled cell bodies in the GPe in both genotypes, confirming that both Npr3+ and ChAT+ GPe neurons project to the amygdala. Together, these findings indicate that pallidocortical neurons project not only to the cortex but also to other subcortical targets, and the distribution of these may differ according to both their molecular identity and their cortical projection target.

Although *Chat* and *Slc32a1* label the majority of pallidocortical neurons, in our dataset, there remained a sizeable population of pallidocortical neurons (36%) that were not labeled by either marker. The Allen Brain Mouse Atlas in situ hybridization data (60) shows a sparse population of glutamatergic neurons at the GPe border (*SI Appendix*, Fig. S6*C*). To determine whether these glutamatergic neurons are pallidocortical projection neurons, we again used pseudotyped RV tracing, with injection of Cre-dependent TVA-GFP helper virus into the GPe of *Vglut2-i-Cre* mice, and pseudotyped (EnvA) RV into either the motor cortex or the insular cortex (*SI Appendix*, Fig. S6 *D* and *E*). We observed Vglut2+ TVA-GFP expression along the ventral, lateral, and medial borders of the GPe. In addition, several of these Vglut2+ GPe neurons were also labeled with RV, suggesting that indeed there is a sparse population of glutamatergic neurons in or surrounding the GPe that project directly to the cortex (*SI Appendix*, Fig. S6 *D* and *E*). Future work will examine whether these are bona fide GPe neurons by examining their innervation by striatal projection neurons.

### Pallidal Neurons Receive Monosynaptic Glutamatergic Input from Motor and Nonmotor Cortical Regions.

**Fig. 4.**
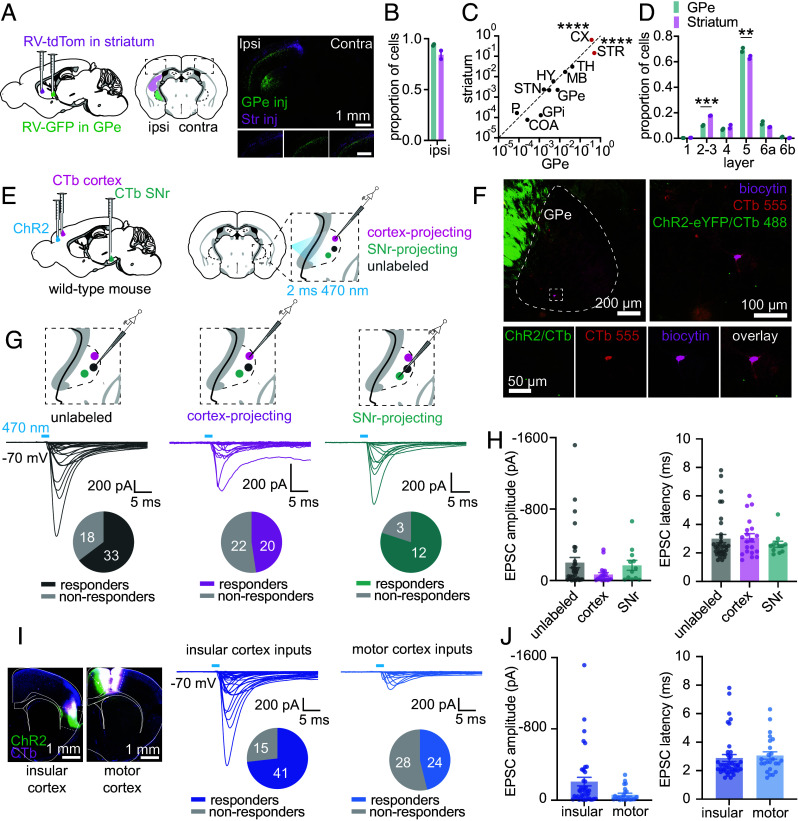
Cortical input to GPe neurons. (*A*) RV retrograde tracing of cortical inputs to the GPe. *Left,* Schematic of the experimental approach. *Right,* Example images of cortex ipsilateral and contralateral to the injection site. *Inset*, zoom of the cortical region. (*B*) Proportion of all cells innervating the GPe and striatum ipsilateral to the injection site (n = 2 mice, mean (±SEM) total cells for GPe = 72,400 (13,563), mean (±SEM) total cells for striatum = 39,834 (1,754). (*C*) Proportion of ipsilateral cells innervating the GPe vs striatum, stratified by broad brain regions (n = 2 mice). There was a significant difference between the GPe and striatum for cortical input (*P* < 0.0001) and striatal input (*P* < 0.0001) after multiple comparison correction (two-way ANOVA with Šídák's multiple comparison test). *Key,* STR: striatum, CX: cortex, TH: thalamus, MB: midbrain, HY: hypothalamus, STN: subthalamic nucleus, GPe: globus pallidus externa, GPi: globus pallidus interna, HPF: hippocampal formation, COA: cortical amygdala area, P: pons. (*D*) Proportion of all ipsilateral cortical cells innervating the GPe vs striatum, stratified by cortical layer (n = 2 mice). There was a significant difference between the GPe and the striatum for layers 2 to 3 (*P* = 0.0001), and layer 5 (*P* = 0.0014) after multiple comparison correction (two-way ANOVA with Šídák's multiple comparison test). (*E*) Schematic for experimental electrophysiological verification of cortical innervation to the GPe in wild-type mice. Cortical axons were stimulated with single 2 ms blue light pulses. (*F*) Example histological image of an acute brain slice used for electrophysiological analyses showing a biocytin and CTb-labeled cortex-projecting neuron analyzed by whole-cell recording. (*G*) Synaptic currents evoked by ChR2 stimulation of cortical axons recording in three different GPe neuron types: unlabeled (n = 51 cells, 14 mice), cortex-projecting (pallidocortical, n = 42 cells, 15 mice), and SNr-projecting (pallidonigral, n = 15 cells, 5 mice). The proportions of responding vs nonresponding cells are shown below the traces. (*H*) Summary of EPSC amplitude and onset latency for all responding neurons. Mean and SEM are shown. (*I*) Example histology of ChR2 and CTb injections in the insular cortex and motor cortex are shown. Synaptic currents evoked by ChR2 stimulation of axons from the insular cortex (n = 56 cells, 10 mice) and the motor cortex (n = 52 cells, 6 mice) to all GPe neuron types. (*J*) Summary of EPSC amplitude and onset latency for all responding neurons. Mean and SEM are shown.

The GPe receives direct synaptic input from the primary and secondary motor cortex, (36, 50) but the extent to which other cortical regions also synapse onto GPe neurons and which GPe neuron subtypes receive this input is not fully understood. Having confirmed that pallidocortical neurons project to both sensorimotor and higher-order association areas of the cortex, we examined whether GPe neurons receive input directly back from nonmotor and higher-order cortical regions. To assess this anatomically, we injected a nonpseudotyped RV encoding a fluorophore into the GPe to generate a cortex-wide map of neurons whose axon terminals innervate the GPe (Fig. 4*A* and *SI Appendix*, Figs. S7 and S8) (62). As a control, we simultaneously injected a similar RV expressing a different fluorophore into the striatum and compared the distribution and pattern of inputs between the two basal ganglia regions. We observed that inputs to the GPe and striatum arose predominantly from neurons in the brain hemisphere ipsilateral to the injection site (Fig. 4*B*). Furthermore, the cortex was the second most common site for input to the GPe, after the striatum (Fig. 4*C* and *SI Appendix*, Fig. S8 *A* and *C*). The mean total number of cortical cells projecting to the GPe was 26,616, and the mean total number of cortical cells projecting to striatum was 25,455. Furthermore, the majority of GPe input from the cortex arises from deep cortical layers, in particular, layer V, whereas input to the striatum arises from both deep and more superficial cortical layers, layers II and III (Fig. 4*D*). Cortical input to the GPe arises from a large swath of cortical regions. The sensorimotor cortex provides the largest input, followed closely by higher-order association areas such as the agranular insular cortex (*SI Appendix*, Fig. S8 *B*–*D*).

We examined whether these anatomical observations indicate the existence of functional synapses. Based on prior studies (50), we hypothesized that the “traditional” prototypic output neurons of the GPe—for example, those projecting to the substantia nigra pars reticulata (SNr)—may receive weaker synaptic input from the cortex compared to other GPe neuron types. To test this, we injected CTb labeled with one fluorophore into the cortex and CTb labeled with a different fluorophore into the SNr of wild-type mice. In addition, we injected a virus encoding the optogenetic neural activator, channelrhodopsin (ChR2), into either the motor or insular cortex (Fig. 4 *E* and *F* to activate cortical axonal terminals in the GPe with blue light in acute brain slices prepared 3 to 8 wk after injection (mean = 4.5 wk, std = 1.2 wk). We recorded from CTb-labeled neurons and nearby unlabeled neurons, within or as close to the terminal field of ChR2-expressing cortical axons as possible.

While collecting these data, we observed a proportion of GPe neurons with unusually fast excitatory currents evoked by pulses of blue light, with a latency of less than 1.5 ms from the onset of the blue light pulse (*SI Appendix*, Fig. S9*A*). We attempted to eliminate these currents using pharmacologic blockers of neuronal spiking (TTX 10 µM) and excitatory glutamatergic synaptic transmission (NBQX, CPP 10 µM), and found that they were not pharmacologically suppressible, consistent with direct activation of optogenetic photocurrents (*SI Appendix*, Fig. S9*A*). Most neurons with photocurrents were pallidocortical neurons (those labeled with CTb injected into the cortex), consistent with retrograde expression of ChR2 from the cortex to the GPe, even in the absence of clear ChR2-associated YFP expression in the GPe. Cells expressing these short-latency currents were excluded from further analyses.

After exclusion of these photocurrents, we were surprised to find that cortical-mediated synaptic currents were evoked in many neurons in the GPe, including those projecting to SNr, as well as randomly selected neurons not labeled with any fluorophore (“unlabeled”) (Fig. 4 *G* and *H*). Importantly, we also identified synaptic input to cortex-projecting neurons, confirming the existence of a reciprocal circuit in which pallidocortical neurons that project to the cortex receive direct excitatory input back from the cortex (Fig. 4*H*). We found that cortical inputs arose from both the motor and insular cortex, confirming that the GPe receives direct synaptic input from motor and nonmotor regions of the cortex (Fig. 4 *I* and *J*). In this dataset from wild-type mice, synaptic currents evoked by insular cortex inputs were more common and larger than motor cortex inputs, but in a second dataset (*Rbp4-Cre* mice) (*SI Appendix*, Fig. S10*B*), insular and motor cortex inputs evoked synaptic currents that were similar in magnitude. Therefore, GPe neurons are innervated by projections from the motor and nonmotor cortex, but the relative contribution of each input is unclear.

### Pallidocortical Neurons Receive Reciprocal Synaptic Input from Cortical Projection Neurons.

**Fig. 5.**
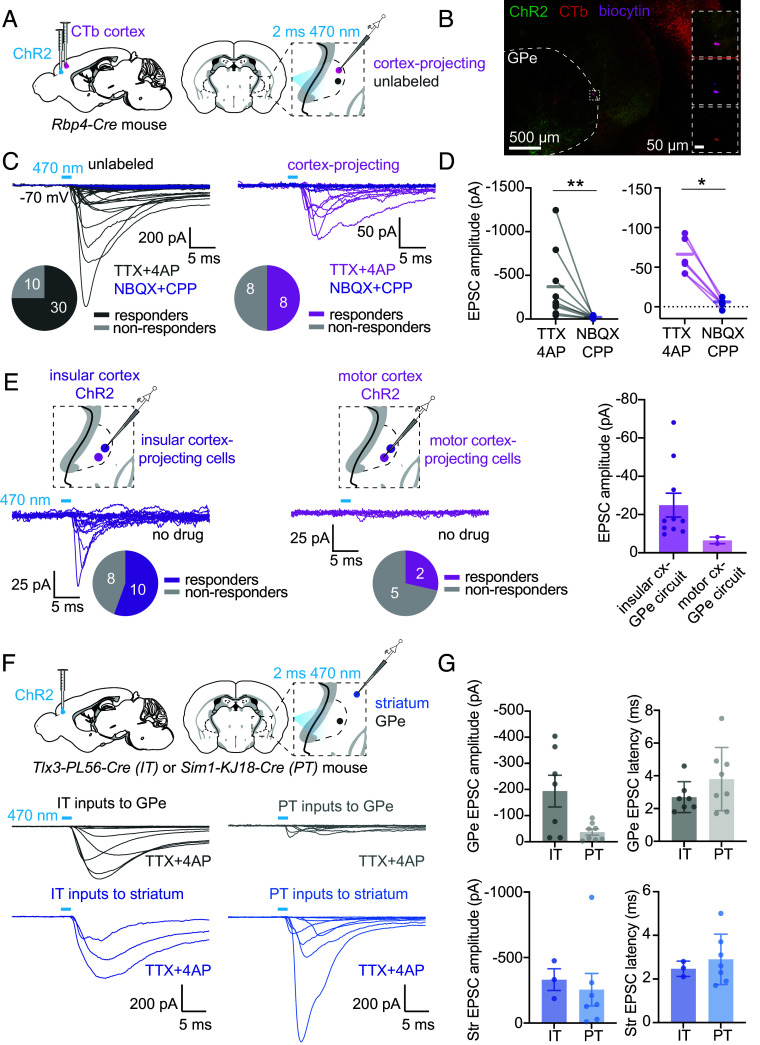
Cell type–specific cortical input to GPe neurons. (*A*) Schematic of the experimental approach. Single 2 ms 470 nm light pulses were used to stimulate *Rbp4*+ cortical axons. Recordings from GPe neurons were made in the presence of TTX and 4AP to isolate monosynaptic inputs, and in the absence or presence of blockers of glutamatergic synaptic transmission. (*B*) Example histological image of an acute brain slice used for electrophysiological recordings showing a biocytin-filled, CTb-labeled cortex-projecting neuron. (*C*) Synaptic currents evoked by ChR2 stimulation of Rbp4+ cortical inputs for unlabeled (n = 40 cells, 11 mice) and cortex-projecting (pallidocortical, n = 16 cells, 8 mice) cells. Responses in the presence of glutamatergic synaptic blockers are shown (purple traces) for a subset of cells. The proportion of responding vs nonresponding cells in the absence of synaptic blockers is shown below the traces. (*D*) Summary of EPSC amplitude for neurons in the absence and presence of glutamatergic synaptic blockers. Mean and SEM are shown. Responses were significantly reduced by synaptic blockers for both unlabeled and cortex-projecting neurons. Unlabeled cells: n = 9 cells, 9 mice: Wilcoxon matched-pairs signed rank test W = 45, *P* = 0.0039; cortex-projecting cells: n = 6 cells, 3 mice: W = 21, *P* = 0.0312. (*E*) Synaptic currents evoked by ChR2 stimulation of Rbp4+ axons (in the absence of TTX and 4AP) from *Left,* insular cortex, recording from insular cortex-projecting GPe neurons (n = 18 cells, 3 mice), and *Middle,* motor cortex, recording from motor cortex-projecting GPe neurons (n = 7 cells, 4 mice). The proportions of responding vs nonresponding cells are shown below the traces. *Right,* Summary of EPSC amplitude for all responding neurons. Mean and SEM are shown. (*F*) Schematic of the experimental approach: ChR2 was injected into the cortex of either *Tlx3-PL56-Cre* mice (to target intratelencephalic, IT, neurons) or *Sim1-KJ18-Cre* mice (to target pyramidal tract, PT, neurons). Synaptic currents evoked by ChR2 stimulation of IT axons in the GPe (n = 15 cells, 2 mice) and striatum (n = 3 cells, 2 mice) and PT axons in the GPe (n = 22 cells, 2 mice) and striatum (n = 9 cells, 2 mice). (*G*) Summary of EPSC amplitudes and onset latency for IT and PT inputs to the GPe and striatum. Mean and SEM are shown.

To eliminate off-target retrograde expression of ChR2 and examine the contribution of different cortical neurons to pallidal synapses, we repeated the same experiments in three different transgenic mouse lines to specifically target deep-layer cortical projection neurons. In an *Rbp4-Cre* mouse (59, 63) (*SI Appendix*, Table S1), which targets layer 5 projection neurons, we again confirmed the presence of excitatory synaptic input to both unlabeled and pallidocortical neurons in the GPe (Fig. 5 *A*–*D*). With this approach, we no longer observed any fast photocurrents (<1.5 ms from light pulse onset). We confirmed that the evoked currents were monosynaptic as they could be evoked in the presence of action potential blockade (TTX 10 µM) together with the potassium channel antagonist, 4AP (400 µM), which recovers synaptic vesicle release in the absence of sodium action potentials (64) (Fig. 5 *C* and *D*). Similarly, the currents were suppressed by blockers of glutamatergic neurotransmission (NBQX, CPP 10 µM), confirming that they are mediated by the excitatory neurotransmitter, glutamate.

We confirmed the presence of topographically reciprocal cortical-pallido-cortical circuits by recording inputs from topographically matched cortical inputs and GPe projections. We found that insular cortex inputs reliably generated excitatory currents in insular cortex-projecting GPe neurons, whereas motor cortex inputs to motor cortex-projecting GPe neurons were sparse (Fig. 5*E* and *SI Appendix*, Fig. S9*B*). This was the case both for *Rbp4-Cre* mice (Fig. 5*E*) and wild-type mice (*SI Appendix*, Fig. S9*B*).

Since Rbp4-positive cortical neurons include both intratelencephalic (IT) projection neurons and pyramidal tract (PT) projection neurons, we narrowed down the source of cortical input to the GPe by repeating the experiments in either *Tlx3-PL56-Cre* mice (59, 63) to target IT neurons or *Sim1-KJ18-Cre* mice to target PT neurons (59, 63) (Fig. 5 *F* and *G* and *SI Appendix*, Fig. S10*D*) (*SI Appendix*, Table S1). As a positive control for variability in ChR2 expression across mice, we also recorded from striatal neurons in the same brain slices, as close as possible to the recorded GPe neurons, and found that whereas *PT*-inputs to the striatum were robust, they were small and infrequent to GPe neurons. In contrast, *IT*-inputs to the striatum and GPe were of similar magnitude (Fig. 5 *F* and *G*). The existence of IT neuron synaptic input to the GPe is surprising, given the result of previously published anatomic studies which showed that projections from the primary and secondary motor cortex to the GPe arise predominantly from PT neurons rather than IT neurons (36). It is possible that this discrepancy results from differences in the properties or representation of IT and PT-type neurons in *Tlx3-PL56-Cre* and *Sim1-KJ18-Cre* mice in different cortical regions or could be due to sampling limitations of our electrophysiological recordings. It is also possible that the IT projection is anatomically sparse but carries strong synaptic weight.

### Cortical Excitatory Input to the GPe can Evoke Changes in GPe Neuron Spiking Activity.

**Fig. 6.**
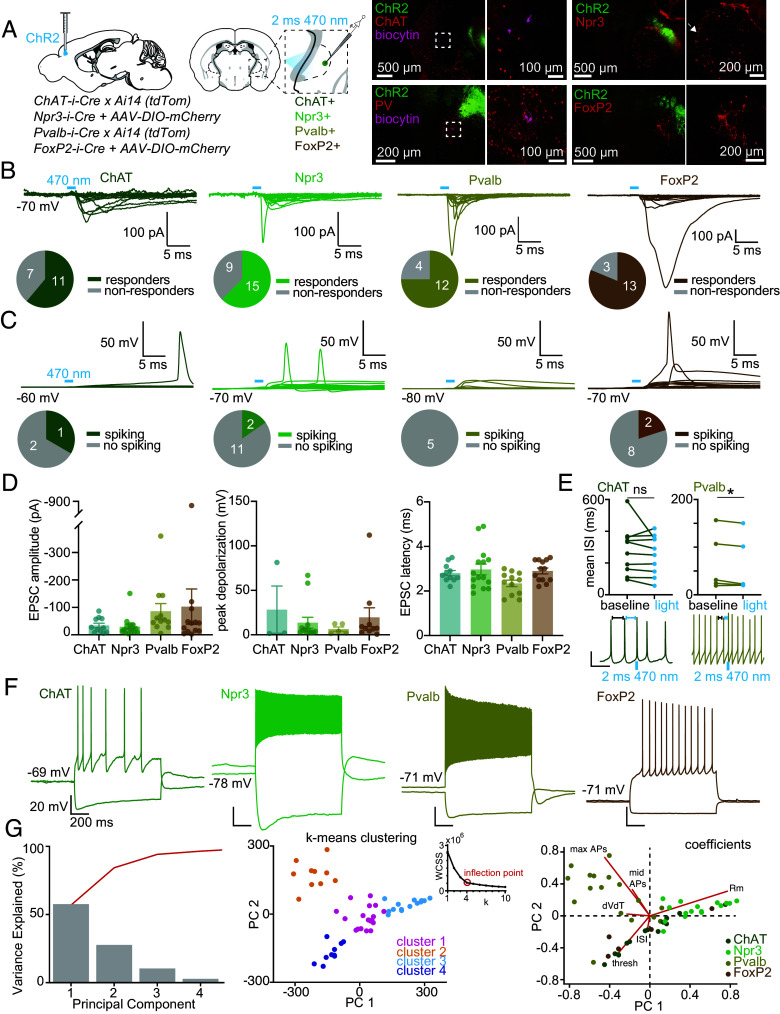
Cortical input to specific cell types in the GPe. (*A*) Schematic of the experimental approach: non-*Cre*-dependent ChR2 was injected into the cortex, a *Cre*-dependent fluorophore labeled different neuron-types (ChAT+, Npr3+, Pvalb+, FoxP2+). 2 ms pulses of 470 nm light were delivered to stimulate cortical inputs to the GPe. Example histological images are shown, biocytin indicates a recorded neuron, arrow highlights Npr3+ pallidocortical neurons located at the lateral borders of the GPe. (*B*) Synaptic currents evoked by ChR2 stimulation of cortical inputs to ChAT+, Npr3+, Pvalb+, and FoxP2+ neurons are shown. The proportion of responding vs nonresponding cells is shown below traces. Number of cells/mice: ChAT+ n = 18 cells, 2 mice; Npr3+ n = 24 cells, 5 mice, Pvalb+ n = 16 cells, 3 mice; FoxP2+ n = 16 cells, 4 mice. (*C*) Synaptic potentials evoked by ChR2 stimulation of cortical inputs to ChAT+, Npr3+, Pvalb+, and FoxP2+ neurons. Proportion of neurons that fired an action potential in response to ChR2 stimulation are shown below the traces. Number of mice/cells: ChAT+ n = 3 cells, 2 mice, Npr3+ n = 19 cells, 5 mice, Pvalb+ n = 5 cells, 2 mice, FoxP2+ n = 12 cells, 4 mice. (*D*) Summary of EPSC amplitude, peak depolarization, and EPSC latency evoked by ChR2 stimulation for all responding neurons. Mean and SEM are shown. (*E*) Mean prelight and postlight ISI was measured in 10 ChAT+ neurons (n = 2 mice) and 5 Pvalb+ neurons (n = 2 mice) that were spontaneously spiking. Traces are shown for each cell type (timing of 470 nm 2 ms light pulse is indicated by the blue bar beneath traces). The scale bar indicates 20 mV, 200 ms. There was no significant difference in ISI after light, on average, for ChAT+ neurons (Two-tailed paired *t* test: t = 1.084, df = 9, *P* = 0.3065). There was a significant difference in ISI after light for Pvalb+ neurons (Two-tailed paired *t* test: t = 2.910, df = 4, *P* = 0.0437). (*F*) Example current clamp recordings from ChAT+, Npr3+, Pvalb+, and FoxP2+ GPe neurons. A hyperpolarizing response and a suprathreshold depolarizing response is shown for each cell. (*G*) Principal components analysis for intrinsic properties of different GPe cell types. *Left,* Scree plot results. Percent variance in the data explained by the first four principal components. *Middle,* K-means clustering results with k = 4 clusters. Data points are color-coded by cluster assignment. The plot illustrates the distribution of data across clusters, n = 52 cells, 14 mice. *Inset:* Elbow plot of Within-Cluster Sum of Squares (WCSS) for different values of k. The maximum of the ratio of the second derivative/first derivative was used to identify the optimal value for k (the inflection point of the curve). *Right,* Biplot of the first two principal components (PC1 and PC2) showing variable coefficients and scores. Data points represent the scores for each cell on PC1 and PC2 and are color-coded according to their genetic identity (ChAT+, Npr3+, Pvalb+, FoxP2+). Red lines indicate the direction and magnitude of variable coefficients, with length proportional to the contribution of each variable to the principal components. *Abbreviations*, thresh: current step to elicit first action potential, dVdT: action potential waveform acceleration, ISI: interspike interval, Rm: membrane resistance, midAPs: number of action potentials at midpoint current step, maxAPs: number of action potentials at maximum current step.

To assess the functional impact of excitatory cortical input on spiking activity of GPe neurons, we recorded from molecularly defined neuron types in the GPe during optogenetic stimulation of cortical axons from either the motor cortex or the insular cortex. We used transgenic mouse lines that permit conditional *Cre-*dependent fluorophore labeling of molecularly defined neurons (*SI Appendix*, Table S1). The neuron subtypes targeted were (40)ChAT-expressing neurons (ChAT+, labeled using *ChAT-i-Cre* transgenic mice crossed to a tdTomato transgenic reporter line, *Ai14*), Npr3-expressing neurons (Npr3+, labeled using virally mediated *Cre*-dependent fluorophore expression in *Npr3-i-Cre* mice, canonical GPe parvalbumin-expressing “prototypic” neurons (Pvalb+, *PV-i-Cre x Ai14* transgenic cross), and FoxP2-expressing “arkypallidal” neurons (40) (FoxP2+, virally mediated *Cre*-dependent fluorophore expression in *FoxP2-i-Cre mice*) (Fig. 6*A*). We recorded excitatory synaptic currents in all neuron types, and these were comparable in magnitude and latency across cell types (Fig. 6 *B* and *D*). When assessing the membrane potential response to this synaptic input in current clamp mode in the same neurons, we found that this input evoked action potentials in many cell types (ChAT+, Npr3+, and FoxP2+) (Fig. 6 *C* and *D*). Although light pulses did not evoke spikes in Pvalb+ neurons that were not spontaneously active, in a subset of spontaneously firing Pvalb+ neurons, we found that light pulses reduced the ISI immediately following the light pulse, effectively resetting the phase of firing (Fig. 6*E*).

To examine whether an unbiased analysis of intrinsic electrophysiological properties segregates the data from all four cell types into clusters according to molecular phenotype, we used principal component analysis (PCA). PCA based on a range of intrinsic electrophysiological properties revealed four distinct clusters, three of which segregated broadly into Pvalb*+-*like, ChAT+-like, or Npr3+-like identities, and a fourth central cluster that contained a mixture of all four cell types (Fig. 6 *F* and *G*). Key electrophysiological features that distinguished the clusters included membrane resistance, ISI, action potential waveform acceleration, current step to generate a first action potential and the maximum firing frequency (Fig. 6*G* and *SI Appendix*, Fig. S12*I*). We also performed a combined PCA of intrinsic electrophysiological properties on the full dataset of GPe neurons, including both the anatomically labeled pallidocortical neurons (Fig. 2) and molecularly labeled GPe neurons (Fig. 6). We found that anatomically labeled pallidocortical neurons overlap in the same quadrants of PCA space with ChAT+ and Npr3+ neurons, suggesting that these neurons share electrophysiologic properties (*SI Appendix*, Fig. S12*J*).

## Discussion

The GPe is a heterogeneous, richly connected basal ganglia nucleus that plays a pivotal role in determining basal ganglia output. Although the function of the GPe during behavior remains mysterious, numerous lines of evidence implicate it in both motor and reward-related aspects of goal-directed behavior, (1–3, 9, 10) and GPe dysfunction has been linked to multiple neurologic and psychiatric disorders including Parkinson’s disease (1, 11, 41, 65), Huntington’s disease (17, 18), dystonia (14–16), Tourette’s syndrome (19), obsessive compulsive disorder, addiction-related behaviors (66, 67) and sleep disorders (13, 68, 69). Canonical models of the basal ganglia describe the GPe as an internal basal ganglia nucleus, receiving input only from the striatum and subthalamic nucleus and relaying that information only to other basal ganglia nuclei. However, several studies reveal the existence of direct projections from the GPe to the cortex (33, 34, 49) and of direct cortical inputs to the GPe (50) in rodents as well as humans (52), highlighting that the GPe is an output nucleus of the basal ganglia that interfaces directly with the cortex.

We profiled the anatomic, molecular, and electrophysiologic properties of pallidocortical circuits. We demonstrate that pallidocortical neurons encircle the borders of the GPe and maintain a topography that reflects the topography of their cortical projection targets. We show that pallidocortical neurons fall into two distinct electrophysiological clusters, segregated by intrinsic and action potential firing properties. Molecularly, pallidocortical neurons also fall into two distinct categories, *Npr3/Slc32a1-*expressing and *Nkx2-1/Chat-*expressing neurons, although we also find a smaller population of glutamatergic (*Vglut2*-expressing) pallidocortical neurons. These molecular markers can be harnessed to target these neurons for further study using conditional viral expression systems in transgenic mice. We also show that the GPe receives reciprocal input from a broad swath of cortical regions, including nonmotor and higher-order association areas. This input arises from deep layers (V/VI) of the cortex, predominantly from Rbp4+ intratelencephalic cortical projection neurons. This contrasts with cortical inputs to the striatum, which arise from both layers II/III and layer V, and from both intratelencephalic and pyramidal tract projection neurons. Cortical input to the GPe is monosynaptic, glutamatergic, and appears to broadly target different types of GPe neurons and circuits, including those that project to traditional targets such as the SNr as well as those that project to the cortex. The latter projection forms a reciprocal pallido-cortical-pallidal circuit. The functional impact of cortical input on GPe neural activity depends upon the molecular and physiologic properties of the GPe neuron, but we found that even small excitatory inputs from the cortex were able to evoke action potentials in some GPe neurons or reset the phase of spontaneously firing neurons.

The GPe is not unique as a basal ganglia nucleus in receiving direct cortical input. In addition to the known cortical projections to the primary input nucleus, the striatum, and the “hyperdirect pathway” from the motor cortex to STN, we and others (70) show that the SNr also receives direct glutamatergic input from both motor and nonmotor cortical regions (*SI Appendix*, Fig. S13). This suggests that fast communication between the cortex and nonstriatal basal ganglia nuclei is not a rare phenomenon, but rather is a basic principle of basal ganglia structure and function. In keeping with current theories of the role of the basal ganglia in integrating both motor- and nonmotor function, we show that both the GPe and the SNr receive cortical input from motor as well as nonmotor associative cortex. Together, these findings suggest that hyperdirect cortical-basal ganglia loops may support the integration of information about internal state and the external environment in the basal ganglia to produce rapid changes in goal-directed behavior.

There are many open questions about how reciprocal pallidocortical/corticopallidal (and corticonigral) loops contribute to the flow of neural activity through the basal ganglia. Single-unit recordings of GPe neurons during stimulation of cortical input to the GPe in vivo will allow us to build models of how cortical input is processed and filtered by different GPe cell types. In addition, to determine how this cortical input alters information flow through entire basal ganglia loops, it will be essential to simultaneously record in downstream structures such as the SNr, thalamus, striatum, and cortex. Other limitations of this study include our focus on four main cortical projection targets and only two main cortical input regions. This was to provide examples of both motor and nonmotor connectivity; however, there are many cortical regions that we have not explored in detail that are also likely to directly influence pallidal function, such as the orbitofrontal cortex and the retrosplenial cortex. Furthermore, it will be important to directly test the function of pallidocortical neurons and corticopallidal inputs in the regulation of behavior and particularly in goal-directed actions.

In conclusion, we confirm and extend the finding of a direct projection from the GPe to the cortex, which tiles across multiple cortical regions, segregates into two distinct electrophysiological and molecular phenotypes and receives direct input back from deep layers of the motor and nonmotor cortex. This provides a view of how the basal ganglia may flexibly modulate cortical function, respond to multimodal cortical input, and produce diverse disease symptoms when disrupted. This may support identification of therapeutic targets for neuromodulation in basal ganglia disorders.

## Materials and Methods

### Mice.

The following mouse lines were used: C57BL6/J (The Jackson Laboratory, 000664); (GENSAT, MGI: 4367067, bred in house); IT-targeting: *Tlx3-PL56-Cre* (GENSAT, MGI: 5311700, bred in house); PT-targeting: *Sim1-KJ18-Cre* (GENSAT, MGI: 4367070, bred in house), *Pvalb-IRES-Cre* (The Jackson Laboratory, 017320); Ai14 (tdTomato *Cre* reporter line, The Jackson Laboratory, 007914), *Pvalb-IRES-Cre* x *Ai14* (bred in house); *ChAT-IRES-Cre (*The Jackson Laboratory, 006410); *ChAT-IRES-Cre* x *Ai14* (bred in house); *FoxP2-IRES-Cre* (The Jackson Laboratory, 030541); *Npr3-IRES2-Cre-D* (The Jackson Laboratory, 031333). All mice were bred on a C57BL/6 J genetic background, and heterozygotes were used. Adult mice of either sex were used. Animals were kept on a 12:12 regular light/dark cycle under standard housing conditions. All animal care and experimental manipulations were performed in accordance with protocols approved by the Harvard Standing Committee on Animal Care, following guidelines described in the US NIH Guide for the Care and Use of Laboratory Animals.

### Fluorescent In Situ mRNA Hybridization (mRNA FISH) Experiments.

FISH experiments were performed as described previously (71, 72). Animals were deeply anesthetized with isoflurane before decapitation. Brains then were rapidly removed, embedded, and frozen in tissue freezing media (Tissue-Tek^®^ O.C.T. Compound) on dry ice. 20 µm coronal sections containing the GPe were prepared on a cryostat microtome (Leica Biosystems, CM1950) and mounted onto SuperFrost Plus glass slides (VWR) at 20-80 µm intervals between slices in a set. Slices were rapidly refrozen once mounted and stored at −80 °C before performing in situ hybridization. Multiplexed fluorescent mRNA in situ hybridization was performed according to the ACDBio RNAscope Fluorescent Multiplex Assay protocols. Briefly, slices were fixed in 4% paraformaldehyde at 4 °C for 15 min and dehydrated through washing steps in ethanol at increasing concentrations (50%, 70%, 100%) before protease digestion (Protease III, 10 min at room temperature). Probes and amplification/detection reagents were applied to the tissue sections and incubated under conditions stated in the detection protocol provided by ACDBio. Sections were counterstained using DAPI provided in the detection reagent kits and mounted in ProLong Gold mounting media (ThermoFisher Scientific P36934). Probes used are listed in *SI Appendix*, Table S1. Images were captured using a fluorescence microscope with structured illumination (Keyence BZ-X710) using 10X air and 60X oil immersion objectives.

### In Vitro Slice Preparation and Electrophysiology Recordings With Optogenetic Stimulation.

Brain slices were obtained from 2- to 5-month-old mice (both male and female) using standard techniques. Mice were anesthetized by isoflurane inhalation and perfused transcardially with ice-cold artificial cerebrospinal fluid (ACSF) containing (in mM) 125 NaCl, 2.5 KCl, 25 NaHCO3, 2 CaCl2, 1 MgCl2, 1.25 NaH2PO4, and 25 glucose (295 mOsm/kg). Brains were then blocked and transferred into a slicing chamber containing ice-cold ACSF. Coronal slices of the GPe were cut at 300 μm thickness with a Leica VT1000s vibratome in ice-cold ACSF, transferred for 10 min to a holding chamber containing choline-based solution [consisting of (in mM): 110 choline chloride, 25 NaHCO3, 2.5 KCl, 7 MgCl2, 0.5 CaCl2, 1.25 NaH2PO4, 25 glucose, 11.6 ascorbic acid, and 3.1 pyruvic acid] at 34 °C then transferred to a secondary holding chamber containing ACSF at 34 °C for 10 min and subsequently maintained at room temperature (20 to 22 °C) until use.

Electrophysiology recordings: Individual brain slices were transferred into a recording chamber, mounted on an upright microscope (Olympus BX51WI) and continuously superfused (2 to 3 ml min^−1^) with ACSF warmed to 32 to 34 °C by passing it through a feedback-controlled in-line heater (SH-27B; Warner Instruments). Cells were visualized through a 60X water-immersion objective with either infrared differential interference contrast optics or epifluorescence to identify cells expressing fluorophores. For whole cell voltage clamp recording, patch pipettes (2 to 4 MΩ) pulled from borosilicate glass (G150F-3, Warner Instruments) were filled with internal solution containing (in mM) 135 CsMeSO3, 10 HEPES, 1 EGTA, 3.3 QX-314 (Cl− salt), 4 Mg-ATP, 0.3 Na-GTP, and 8 Na2-phosphocreatine (pH 7.3 adjusted with CsOH; 295 mOsm kg^−1^). To record ChR2-activated EPSCs, the membrane voltages were clamped at –70 mV. Slice ChR2 fibers were stimulated with 473 nm laser light, delivered at 6mW for 3 ms using full-field illumination through the objective at 10 s intervals. Whole cell current clamp was used to study intrinsic properties of GPe neurons and their membrane potential responses to ChR2 stimulation. For current clamp recordings, patch pipettes were filled with internal solution containing (in mM) 135 KMeSO3, 10 HEPES, 1 EGTA, 4 Mg-ATP, 0.4 Na-GTP, and 8 Na2-phosphocreatine (pH 7.3 adjusted with KOH; 295 mOsm kg^−1^). At the resting membrane potential, currents were injected for 1,000 ms at 50pA steps from −100 to 1,000 pA. Optogenetic and current injection-induced signals were acquired with a MultiClamp 700B amplifier (Molecular Devices) and digitized at 10 kHz with a National Instruments data acquisition device (NI USB- 6343). Data were recorded and analyzed using a custom program written for MATLAB.

Pharmacologic manipulations: All recordings (unless specified) were performed in the presence of gabazine (10 µM, Tocris) to block GABAergic neurotransmission. Additional pharmacologic manipulations were performed as described in the main text using TTX (10 µM, Tocris), 4AP (400 µM, Sigma-Aldrich), NBQX (10 µM, Tocris), and CPP (µM, Sigma-Aldrich). For post hoc histologic visualization of recorded cells, biocytin 1 mg/mL (Sigma-Aldrich) was added to the internal solution. Slices were fixed in chilled 4% PFA, and histology was performed as described above.

### Analysis.

#### Statistical tests.

Data were tested for normality with the Shapiro–Wilk test. Datasets that were not normally distributed were analyzed with nonparametric statistical tests, including the Kruskal–Wallis test (for > 2 groups) or the Mann–Whitney test (unpaired data) or Wilcoxon test (paired data) for comparisons between two groups. Normally distributed data were analyzed with parametric tests including one-way ANOVA (for > 2 groups) or unpaired or paired t-tests. Mean and SEM are shown for all datasets. *P* values are represented by the following symbols: * for 0.01 < *P* < 0.05, ** for 0.001 < *P* < 0.01, *** for 0.0001 < *P* <0.001, and **** for *P* < 0.0001. See *SI Appendix*, Table S2, Statistical Tests for further details of individual statistical comparisons.

## Supplementary Material

Appendix 01 (PDF)

## Data Availability

All study data are included in the article and/or *SI Appendix*. Additional methods and details of materials are provided in the *SI Appendix*. Data files for each figure and for cortex-wide rabies tracing can be found at https://doi.org/10.7910/DVN/I1USMV (62).
