# Supplementary material for: Reciprocal projections between the globus pallidus externa and cortex span motor and nonmotor regions
Source: Proc Natl Acad Sci U S A. 2025 Jun 3;122(23):e2423367122. doi: 10.1073/pnas.2423367122 (PMC12167972; doi:10.1073/pnas.2423367122)
Supplement: Supplementary file 1 — Appendix 01 (PDF) [file pnas.2423367122.sapp.pdf]

PNAS

Supporting Information for
Reciprocal projections between the globus pallidus externa and cortex span motor and nonmotor regions

Emily A. Ferenczi[1,2], Wengang Wang[1], Anushka Biswas[1], Trent Pottala[1], Yihuan Dong[1], Alison K. Chan[1], Madeline A. Albanese[1], Raina S. Sohur[1], Tingying Jia[3], Kevin J. Mastro[1], Bernardo L. Sabatini[1]

Affiliations
[1]Howard Hughes Medical Institute, Department of Neurobiology, Harvard Medical School, Boston MA 02115
[2]Department of Neurology, Massachusetts General Hospital, Harvard Medical School, Boston, MA 02114
[3]Princeton Neuroscience Institute, Princeton University, 40 Woodlands Wy, Princeton, NJ 08540

Corresponding author
Bernardo Sabatini
Email: bernardo_sabatini@hms.harvard.edu

This PDF file includes:

Figures S1 to S13
Legends for Figures S1 to S13
Supplementary methods
Tables S1 to S3
Supplementary References

Fig. S1

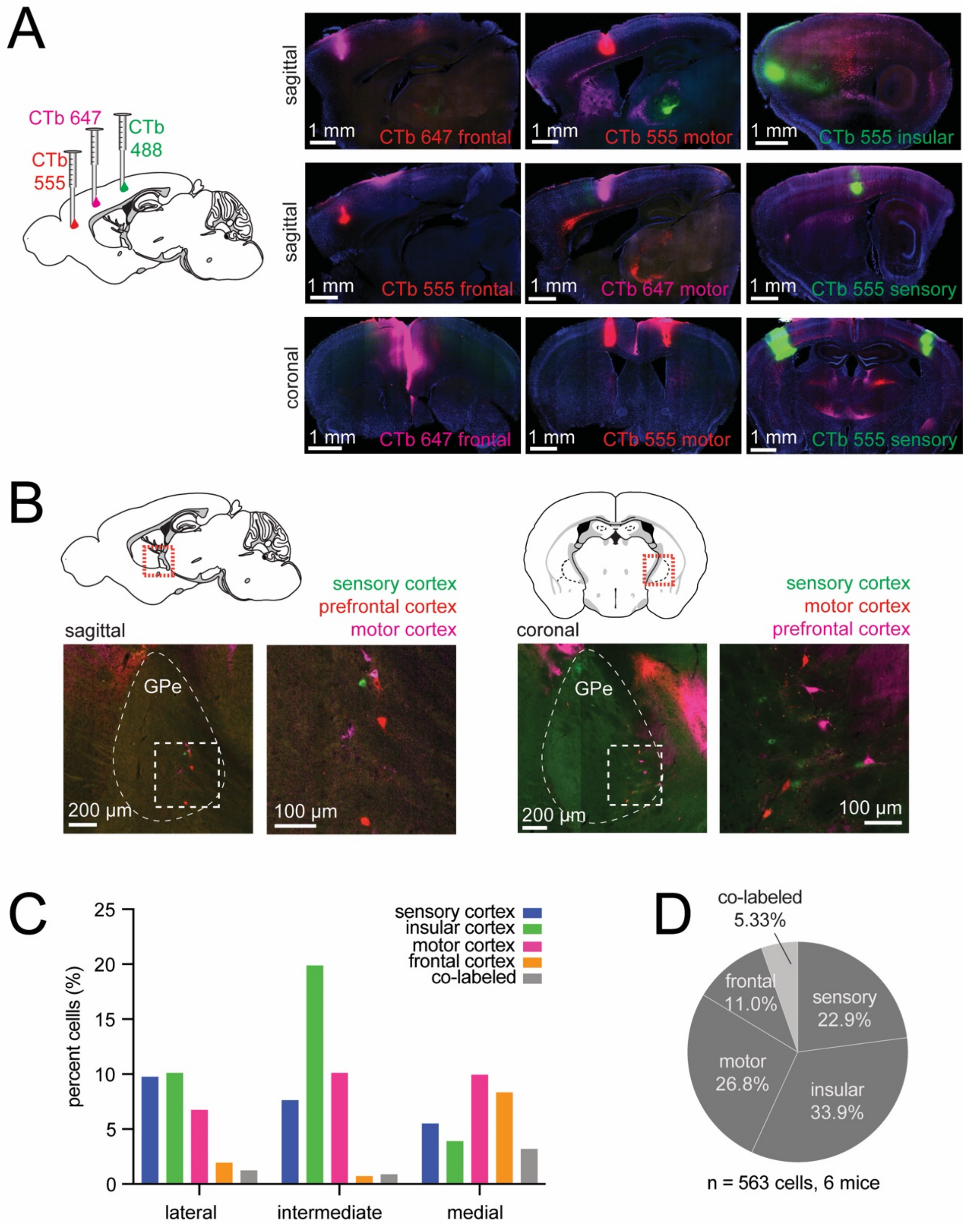
A
CTb 647
CTb 488
CTb 555
sagittal
sagittal
coronal
1 mm
CTb 647 frontal
CTb 555 motor
CTb 555 insular
CTb 555 frontal
CTb 647 motor
CTb 555 sensory
CTb 647 frontal
CTb 555 motor
CTb 555 sensory
B
sensory cortex
prefrontal cortex
motor cortex
sagittal
GPe
200 µm
100 µm
sensory cortex
motor cortex
prefrontal cortex
coronal
GPe
200 µm
100 µm
C
percent cells (%)
0
5
10
15
20
25
sensory cortex
insular cortex
motor cortex
frontal cortex
co-labeled
lateral
intermediate
medial
D
co-labeled 5.33%
frontal 11.0%
sensory 22.9%
motor 26.8%
insular 33.9%
n = 563 cells, 6 mice

Figure S1.

Retrograde labeling of pallidocortical neurons using triple injections of cholera toxin b (CTb).

A, *left,* Schematic of experimental approach: CTb was injected into 3 different cortical areas in each mouse (combinations of sensory cortex, motor cortex, insular cortex, frontal cortex). *right,* Example sagittal (*top, middle rows)* and coronal (*bottom row*) images of CTb injection sites in cortex.

B, Example sagittal and coronal images of CTb labeling in the GPe.

C, Percent of pallidocortical neurons in the GPe from each cortical injection, according to sagittal plane (lateral GPe: ML 2.72 mm, intermediate GPe: ML 2.35 mm, medial GPe: ML 1.95 mm). Co-labeled cells are those in which two different CTb fluorophores were co-expressed.

D, Percent of total number of pallidocortical neurons in the GPe from each cortical injection (total n=563 cells, across 6 mice).

Fig. S2

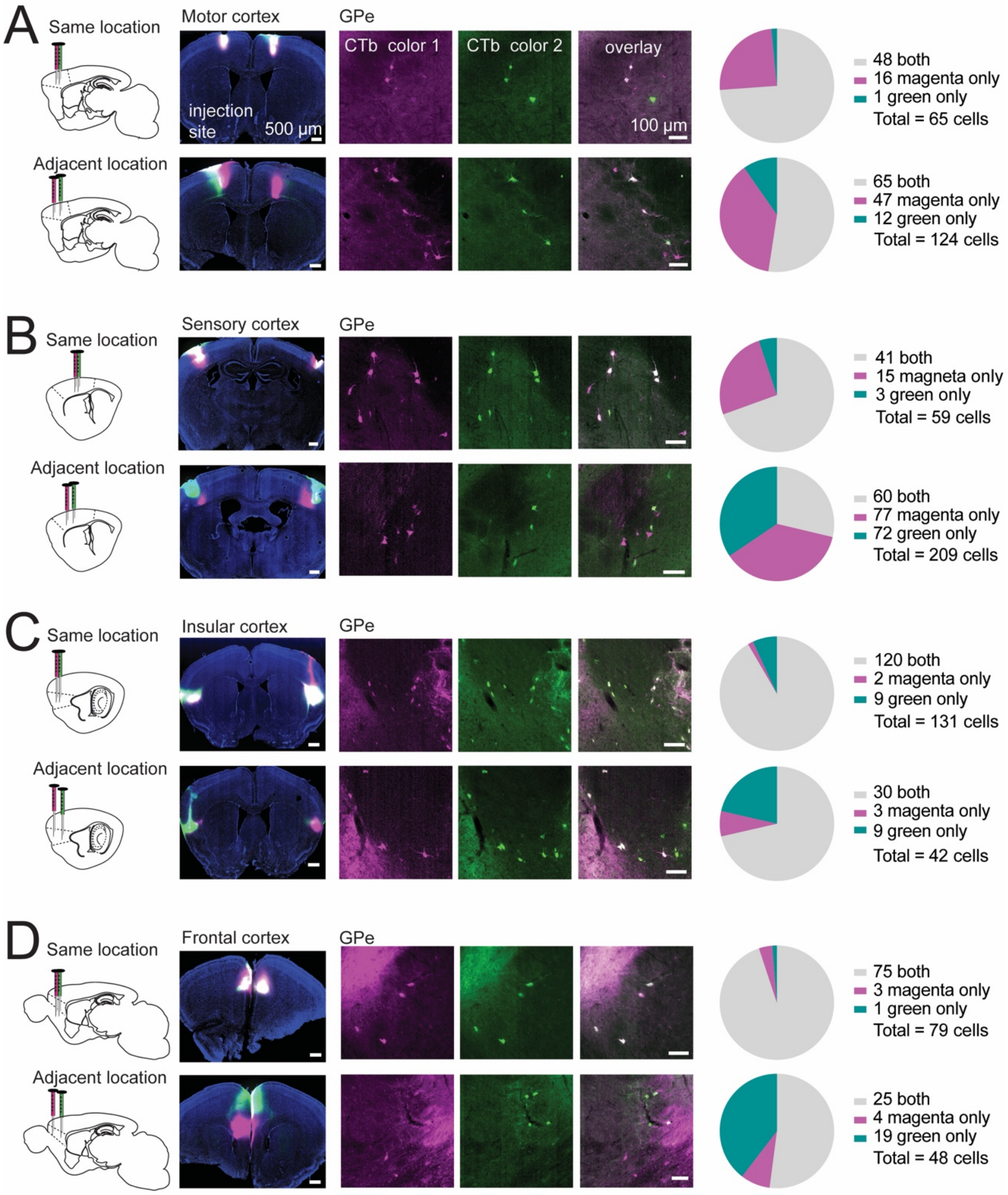
A
Same location
Adjacent location
Motor cortex
GPe
CTb color 1
CTb color 2
overlay
injection site
500 µm
100 µm
48 both
16 magenta only
1 green only
Total = 65 cells
65 both
47 magenta only
12 green only
Total = 124 cells
B
Same location
Adjacent location
Sensory cortex
GPe
41 both
15 magneta only
3 green only
Total = 59 cells
60 both
77 magenta only
72 green only
Total = 209 cells
C
Same location
Adjacent location
Insular cortex
GPe
120 both
2 magenta only
9 green only
Total = 131 cells
30 both
3 magenta only
9 green only
Total = 42 cells
D
Same location
Adjacent location
Frontal cortex
GPe
75 both
3 magenta only
1 green only
Total = 79 cells
25 both
4 magenta only
19 green only
Total = 48 cells

Figure S2.

Retrograde labeling of pallidocortical neurons using double injections of CTb.

A, *left, Schematic of* experimental approach: 2 different CTb fluorophores were injected into motor cortex, *top row,* at the same location (identical injection coordinates), or *bottom row,* at adjacent locations (slightly separated injection coordinates, please see *SI Appendix,* Table S1, Stereotactic Injections for details). *middle,* Example images of injection sites and CTb-labeled neurons in the GPe (Color 1 (magenta pseudocolor)=CTb 657 or CTb 555, color 2 (green pseudocolor)=CTb 488). *right,* Quantification of co-labeling by the two different CTb fluorophores.

B, Same as **A**, for sensory cortex.

C, Same as **A**, for insular cortex.

D, Same as **A**, for frontal cortex.

Fig. S3

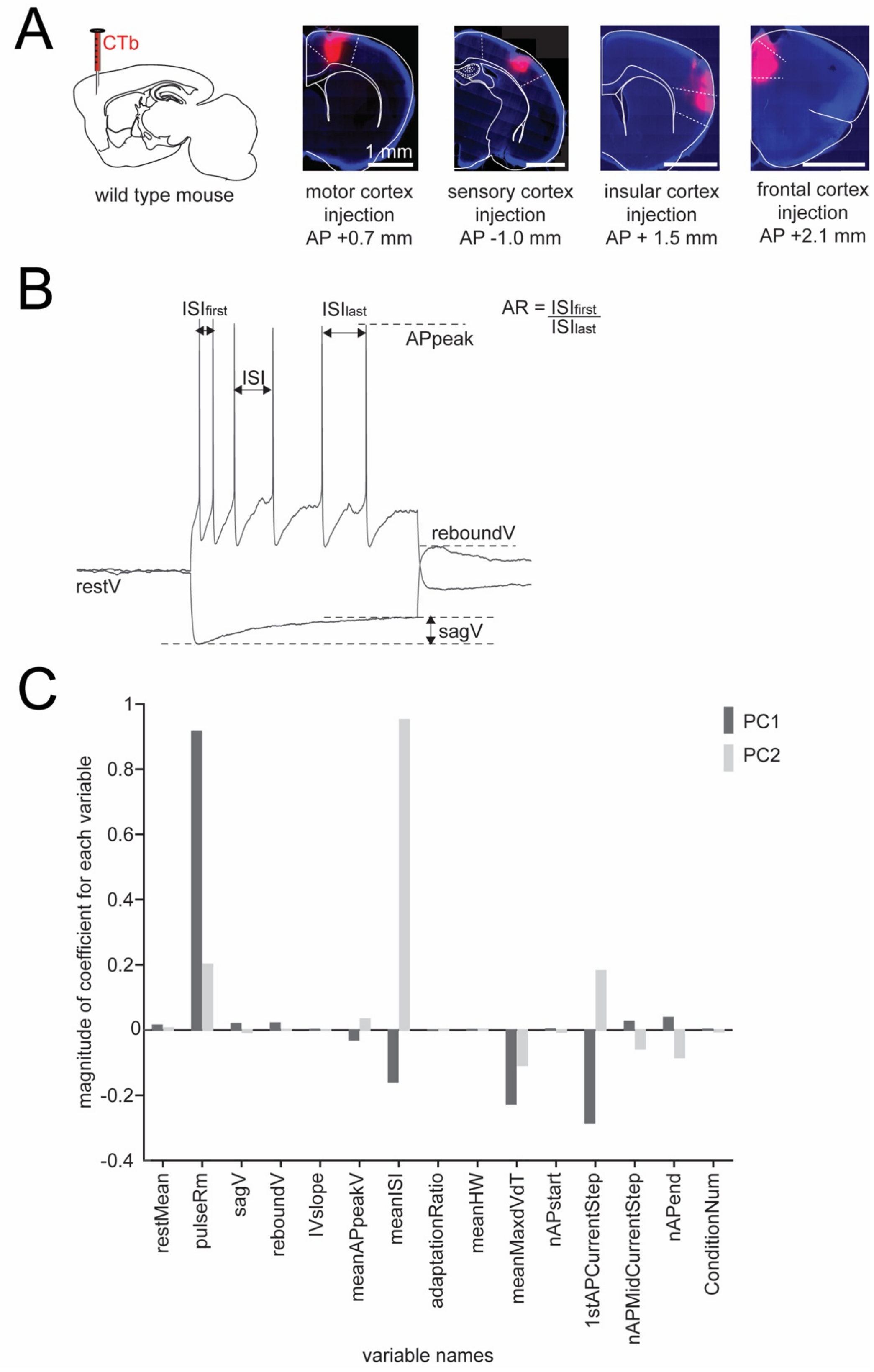

A
CTb
wild type mouse
1 mm
motor cortex injection AP +0.7 mm
sensory cortex injection AP -1.0 mm
insular cortex injection AP + 1.5 mm
frontal cortex injection AP +2.1 mm
B
ISIfirst
ISIlast
ISI
AR = ISIfirst / ISIlast
APpeak
reboundV
restV
sagV
C
PC1
PC2
magnitude of coefficient for each variable
1
0.8
0.6
0.4
0.2
0
-0.2
-0.4
restMean
pulseRm
sagV
reboundV
IVslope
meanAPpeakV
meanISI
adaptationRatio
meanHW
meanMaxdVdT
nAPstart
1stAPCurrentStep
nAPMidCurrentStep
nAPend
ConditionNum
variable names

Figure S3.

Intrinsic properties of pallidocortical neurons.

A, *left,* Schematic of experimental approach: CTb injections into cortex. *right,* Example images of CTb fluorescence in cortex at different injection sites.

B, Diagram of measurements for intrinsic and active membrane properties (see abbreviations below).

C, Bar chart of variable coefficients from PCA. The bar chart shows the coefficients of the variables for the first and second principal components. The x-axis lists the variables, and the y-axis indicates the magnitude of their coefficients. The height of each bar represents the strength and direction of each variable's contribution to the principal component; taller bars signify larger coefficients and greater influence. Positive coefficients are shown above the x-axis, while negative coefficients extend below it.

Variable name abbreviations, restMean: resting membrane potential, pulseRm: membrane resistance, sagV: sag potential, reboundV: rebound potential, IVslope: slope of subthreshold current-voltage relationship, meanAPpeakV: mean peak voltage of all action potentials, meanISI: mean interspike interval between all action potentials, adaptationRatio: mean ratio between the first action potential pair and last action potential pair for each current step, meanHW: mean action potential half-width, meanMaxdVdT: mean maximum rate of change of membrane potential during rising phase of action potential (action potential waveform acceleration), nAPstart: number of action potentials during first current step to elicit an action potential, 1stAPCurrentStep: magnitude of current step that first elicits an action potential, nAPMidCurrentStep: number of action potentials elicited during a current step midway through the current step protocol, nAPend: number of action potentials elicited during the last current step (typically 280-400pA), conditionNum: condition number refers to the experimental group which indicates the cortical target of the recorded pallidocortical neuron.

Fig. S4

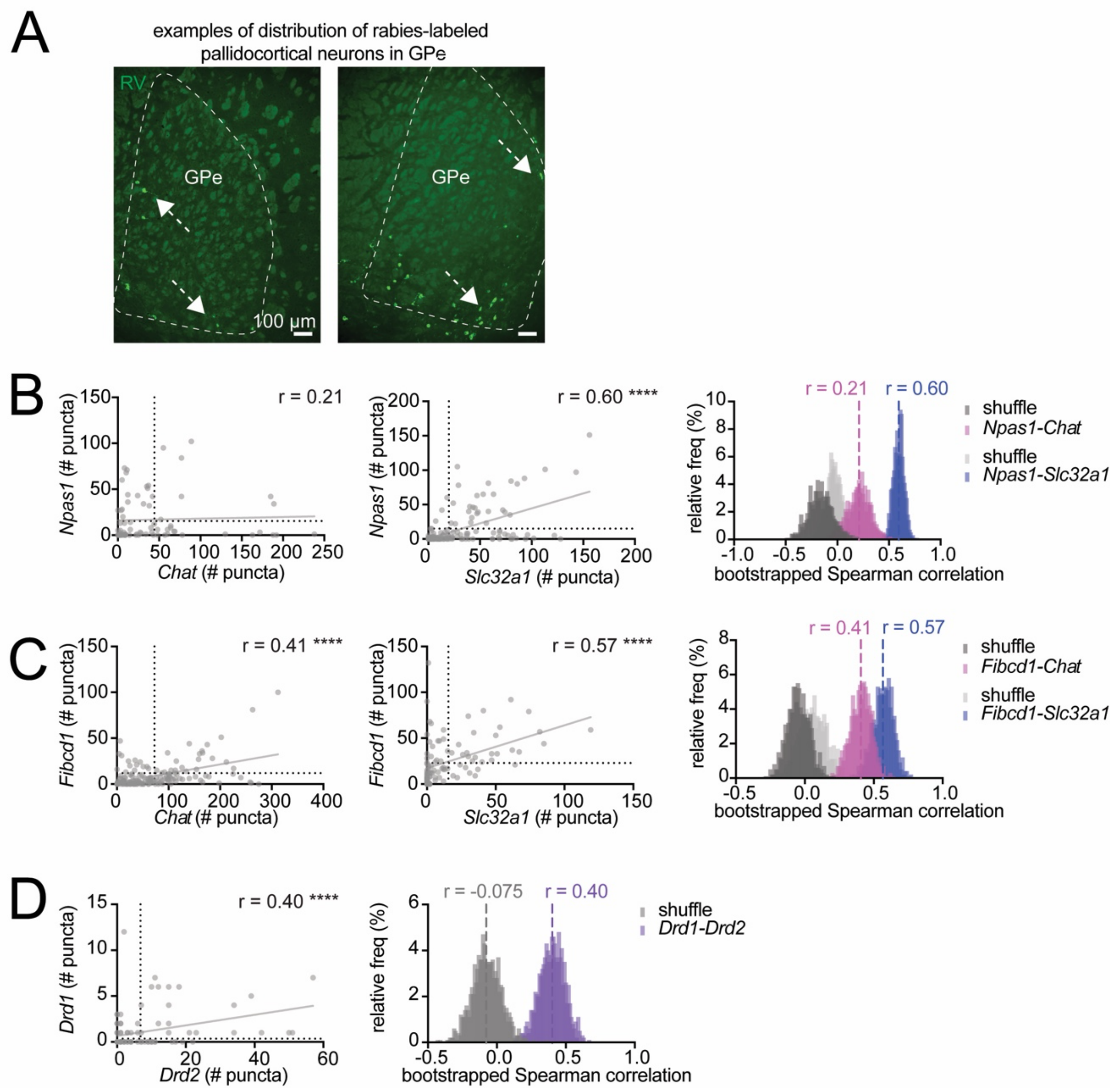

A
examples of distribution of rabies-labeled pallidocortical neurons in GPe
RV
GPe
GPe
100 µm
B
r = 0.21
Npas1 (# puncta)
Chat (# puncta)
r = 0.60 ****
Npas1 (# puncta)
Slc32a1 (# puncta)
r = 0.21
r = 0.60
shuffle
Npas1-Chat
shuffle
Npas1-Slc32a1
relative freq (%)
bootstrapped Spearman correlation
C
r = 0.41 ****
Fibcd1 (# puncta)
Chat (# puncta)
r = 0.57 ****
Fibcd1 (# puncta)
Slc32a1 (# puncta)
r = 0.41
r = 0.57
shuffle
Fibcd1-Chat
shuffle
Fibcd1-Slc32a1
relative freq (%)
bootstrapped Spearman correlation
D
r = 0.40 ****
Drd1 (# puncta)
Drd2 (# puncta)
r = -0.075
r = 0.40
shuffle
Drd1-Drd2
relative freq (%)
bootstrapped Spearman correlation

Figure S4.

mRNA *in situ* characterization of pallidocortical neurons.

A, Examples images (10x magnification) of 20 μm thick brain slices used for mRNA *in situ* hybridization. Arrows indicate location of rabies virus-labeled cells (green), predominantly at the borders of GPe.

B, Quantification of mRNA puncta for *Npas1* vs *Chat* (Spearman rho r=0.205, p=0.072, n=78 cells, 2 mice) and *Npas1* vs *Slc32a1* (Spearman rho r=0.60, $p < 0.0001$, n=197 cells, 2 mice*). right,* Bootstrap analysis for Spearman correlation coefficients for non-shuffled and shuffled data for *Npas1* vs *Chat* and *Npas1* vs *Slc32a1;* mean correlation coefficients for non-shuffled data are shown, along with distribution of shuffled and non-shuffled bootstrapped data.

C, Quantification of mRNA puncta for *Fibcd1* vs *Chat* (Spearman rho r=0.41, $p < 0.0001$, n=158 cells, 2 mice) and *Fibcd1* vs *Slc32a1* (Spearman rho r=0.57, $p < 0.0001$, n=101 cells, 2 mice). Bootstrap analysis for Spearman correlation coefficients for non-shuffled and shuffled data for *Fibcd1* vs *Chat* and *Fibcd1* vs *Slc32a1;* mean correlation coefficients for non-shuffled data are shown, along with distribution of shuffled and non-shuffled bootstrapped data. **c.** Quantification of mRNA puncta for *Drd1* vs *Drd2* (Spearman rho r=0.40, $p < 0.0001$, n=98 cells, 1 mouse). Bootstrap analysis for Spearman correlation coefficients for non-shuffled and shuffled data for *Drd1* vs *Drd2;* mean correlation coefficients for non-shuffled data are shown, along with distribution of shuffled and non-shuffled bootstrapped data.

Fig. S5

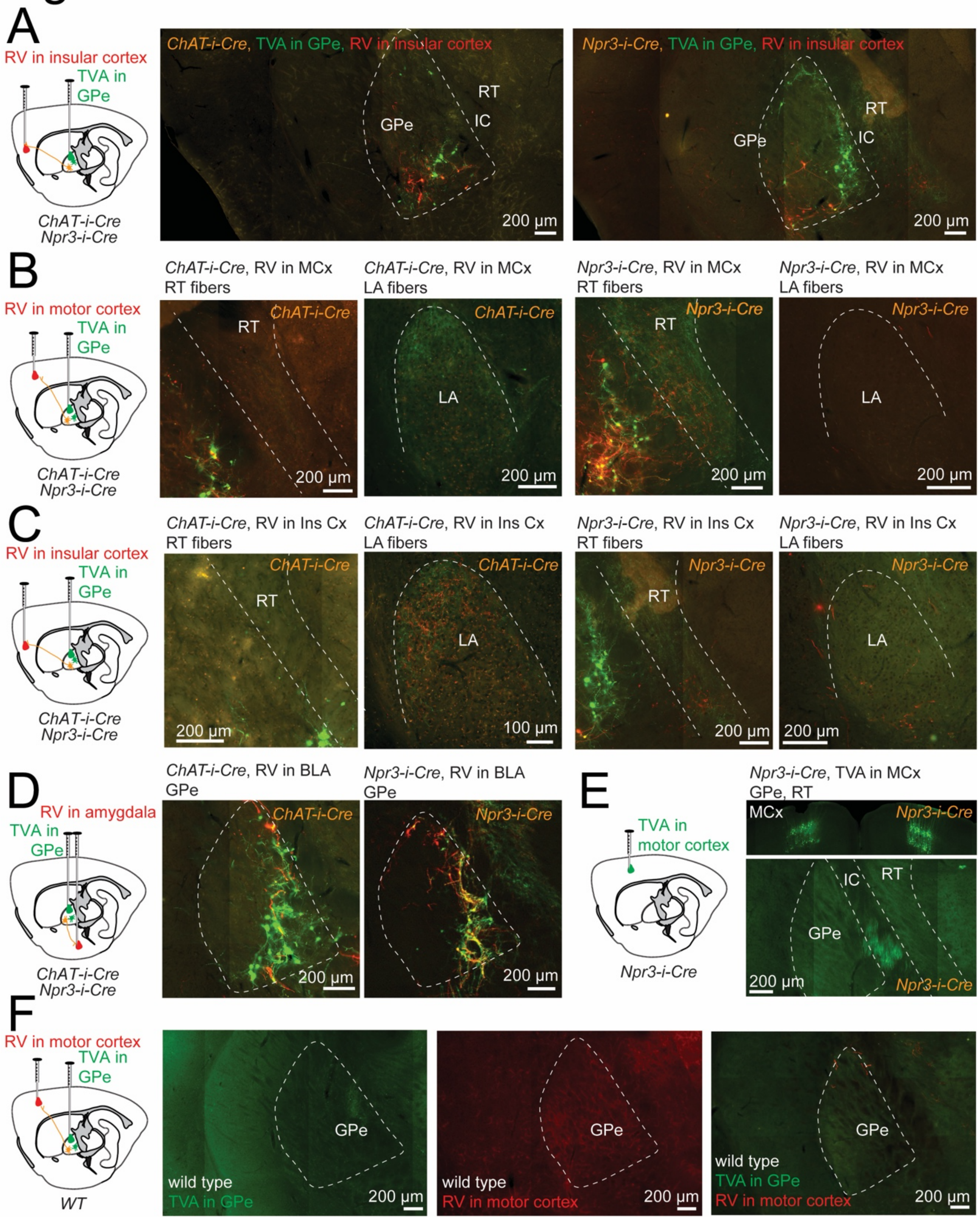

A
RV in insular cortex
TVA in GPe
ChAT-i-Cre
Npr3-i-Cre
ChAT-i-Cre, TVA in GPe, RV in insular cortex
GPe
RT
IC
200 µm
Npr3-i-Cre, TVA in GPe, RV in insular cortex
B
RV in motor cortex
ChAT-i-Cre, RV in MCx
RT fibers
ChAT-i-Cre, RV in MCx
LA fibers
Npr3-i-Cre, RV in MCx
RT fibers
Npr3-i-Cre, RV in MCx
LA fibers
LA
C
ChAT-i-Cre, RV in Ins Cx
RT fibers
ChAT-i-Cre, RV in Ins Cx
LA fibers
Npr3-i-Cre, RV in Ins Cx
RT fibers
Npr3-i-Cre, RV in Ins Cx
LA fibers
100 µm
D
RV in amygdala
ChAT-i-Cre, RV in BLA
GPe
Npr3-i-Cre, RV in BLA
GPe
E
TVA in motor cortex
Npr3-i-Cre, TVA in MCx
GPe, RT
MCx
F
WT
wild type
TVA in GPe
RV in motor cortex

Figure S5.

Collateral targets of pallidocortical neurons

A, Confirmation that *Chat* and *Npr3* can be used to label insular cortex-projecting pallidocortical neurons in the GPe. Experimental approach diagram illustrates *Cre*-dependent TVA-GFP helper virus injection into the GPe and CVS-N2c-dG-mCherry (EnvA) (rabies virus, RV) into the insular cortex. Example images of TVA-expressing (green) and rabies-expressing pallidocortical neurons (red/yellow) in the GPe in *Chat-i-Cre* (*left*) and *Npr3-i-Cre* (*right*) mice.

B, Example images of subcortical collateral fibers from motor cortex-projecting pallidocortical neurons. Experimental approach diagram illustrates *Cre*-dependent TVA-GFP helper virus injection into the GPe and CVS-N2c-dG-mCherry (EnvA) (rabies virus, RV) into motor cortex. Example images of the reticular nucleus of the thalamus (RT) and the lateral amygdala (LA) showing TVA-expressing (green) and/or rabies-expressing axons (red) in *Chat-i-Cre (left two images)* and *Npr3-i-Cre* (*right two images)* mice.

C, Example images of subcortical collateral fibers from insular cortex-projecting pallidocortical neurons. Experimental approach diagram illustrates *Cre*-dependent TVA-GFP helper virus injection into the GPe and CVS-N2c-dG-mCherry (EnvA) (rabies virus) into the insular cortex. Example images of the reticular nucleus of the thalamus (RT) and the lateral amygdala (LA) showing TVA-expressing (green) and/or rabies-expressing axons (red) in *Chat-i-Cre (left two images)* and *Npr3-i-Cre* (*right two images)* mice.

D, Control experiment to confirm the existence of projections from the GPe to the amygdala. Experimental approach diagram illustrates Cre-dependent TVA-GFP helper virus injection into the GPe and CVS-N2c-dG-mCherry (EnvA) (rabies virus) into the amygdala. Example images of TVA-expressing (green) and rabies-expressing pallidocortical neurons (red/yellow) in the GPe in *Chat-i-Cre* (*left*) and *Npr3-i-Cre* (*right*) mice.

E, Control experiment to confirm that Npr3+ neurons in cortex do not send axonal projections directly to the reticular nucleus of the thalamus (RT). Experimental approach diagram illustrates Cre-dependent TVA-GFP helper virus injection into the motor cortex. Example images of TVA-GFP expression in Npr3+ neurons in the motor cortex (*upper image)* and downstream axonal projections in the internal capsule but not the RT (*lower image*).

F, Examples of wild-type control images: *left*, Cre-dependent TVA-GFP helper virus injection was injected into the GPe of wild type mice; *middle*, CVS-N2c-dG-mCherry

(EnvA) (rabies virus) was injected into the motor cortex of wild type mice; *right,* Cre-dependent TVA-GFP helper virus injection was injected into the GPe and CVS-N2c-dG-mCherry (EnvA) (rabies virus) was injected into the motor cortex of wild type mice.

Fig. S6

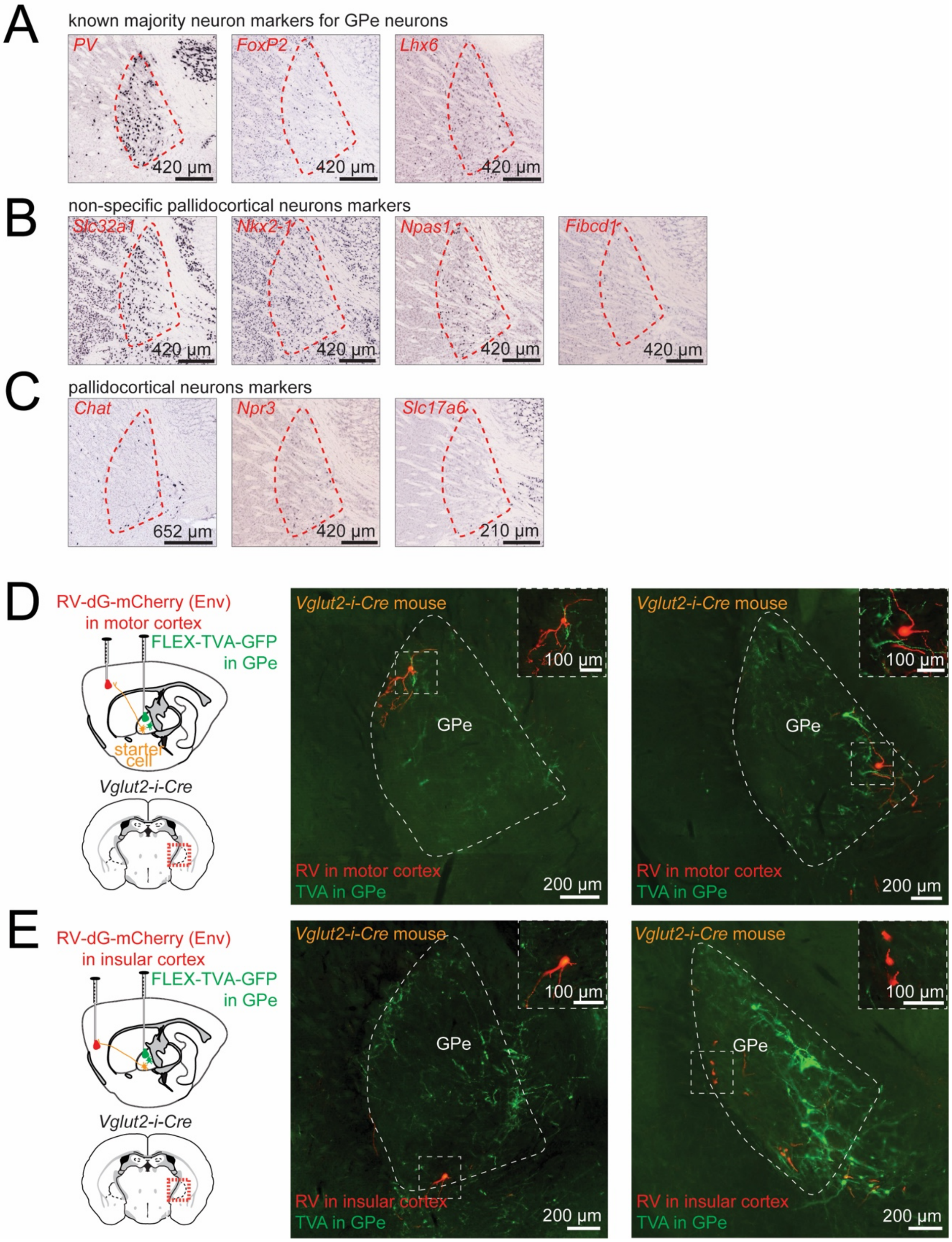

A
known majority neuron markers for GPe neurons
PV
FoxP2
Lhx6
420 μm
B
non-specific pallidocortical neurons markers
Slc32a1
Nkx2-1
Npas1
Fibcd1
420 μm
C
pallidocortical neurons markers
Chat
Npr3
Slc17a6
652 μm
420 μm
210 μm
D
RV-dG-mCherry (Env)
in motor cortex
FLEX-TVA-GFP
in GPe
starter cell
Vglut2-i-Cre
Vglut2-i-Cre mouse
GPe
100 μm
RV in motor cortex
TVA in GPe
200 μm
E
RV-dG-mCherry (Env)
in insular cortex
FLEX-TVA-GFP
in GPe
Vglut2-i-Cre
RV in insular cortex

Figure S6.

Allen Brain Atlas *in situ* markers for GPe neurons and *Vglut2* rabies tracing in GPe

A, Allen Brain Atlas in situ hybridization data[1] for markers of known majority GPe neurons.

Pvalb - https://mouse.brain-map.org/experiment/show/100055042

FoxP2 - https://mouse.brain-map.org/experiment/show/68918262

Lhx6 - https://mouse.brain-map.org/experiment/show/70920033

B, Allen Brain Atlas in situ hybridization data[1] for non-specific markers for pallidocortical neurons.

Slc32a1 - https://mouse.brain-map.org/experiment/show/79677349

Nkx2-1 - https://mouse.brain-map.org/experiment/show/70445269

Npas1 - https://mouse.brain-map.org/experiment/show/74821618

Fibcd1 - https://mouse.brain-map.org/experiment/show/69672462

C, Allen Brain Atlas in situ hybridization data[1] for more specific markers for pallidocortical neurons.

Chat - https://mouse.brain-map.org/experiment/show/253

Npr3 - https://mouse.brain-map.org/experiment/show/70546287

Slc17a6 - https://mouse.brain-map.org/experiment/show/71724696

D, Confirmation that *Vglut2* can be used to label motor cortex-projecting pallidocortical neurons in the GPe. Experimental approach diagram illustrates *Cre*-dependent TVA-GFP injection into the GPe and CVS-N2c-dG-mCherry (EnvA) (rabies virus) into the motor cortex. Two example images of TVA-expressing (green) and rabies-expressing pallidocortical neurons (red/yellow) in the GPe in *Vglut2-i-Cre* mice.

E, Confirmation that *Vglut2* can be used to label insular cortex-projecting pallidocortical neurons in the GPe. Experimental approach diagram illustrates *Cre*-dependent TVA-GFP injection into the GPe and CVS-N2c-dG-mCherry (EnvA) (rabies virus) into the insular cortex. Two example images of TVA-expressing (green) and rabies-expressing pallidocortical neurons (red/yellow) in GPe in *Vglut2-i-Cre* mice.

Fig. S7

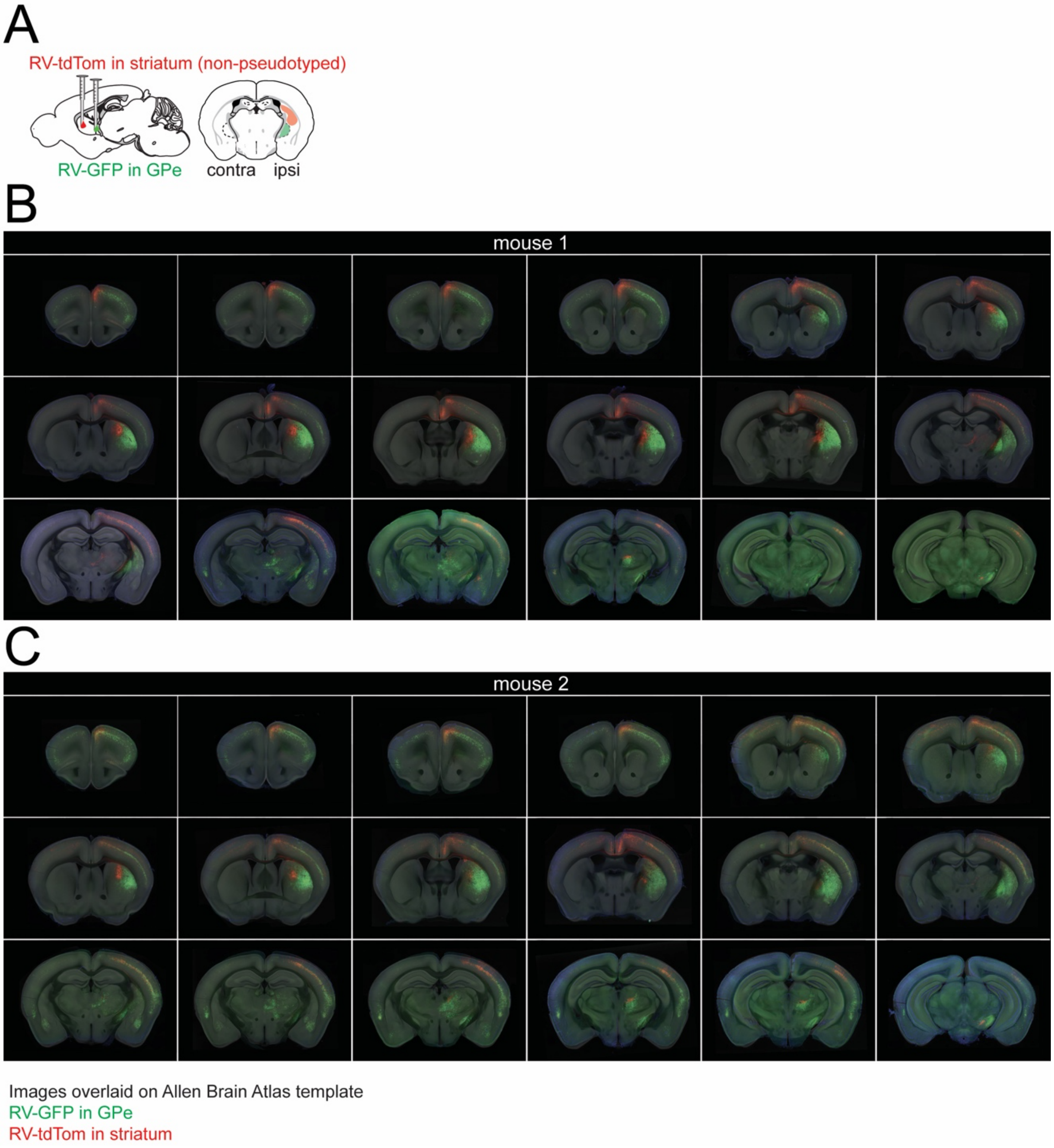
A
RV-tdTom in striatum (non-pseudotyped)
RV-GFP in GPe
contra
ipsi
B
mouse 1
C
mouse 2
Images overlaid on Allen Brain Atlas template
RV-GFP in GPe
RV-tdTom in striatum

Figure S7.

Rabies tracing of corticopallidal versus corticostriatal inputs

A, Experimental approach diagram illustrates injection of non-pseudotyped rabies virus into the GPe (CVS-N2c-dG-GFP) and striatum (CVS-N2c-dG-tdTom) in the same mouse.

B, Example images from mouse 1 showing rabies virus expression across the brain, seven days after CVS-N2c-dG-GFP injection into the GPe (green) and CVS-N2c-dG-tdTom into the striatum (red). Histological images are overlaid on Allen Brain Atlas template.

C, Example images from mouse 2 showing rabies virus expression across the brain, seven days after CVS-N2c-dG-GFP injection into the GPe (green) and CVS-N2c-dG-tdTom into the striatum (red). Histological images are overlaid on Allen Brain Atlas template.

Fig. S8

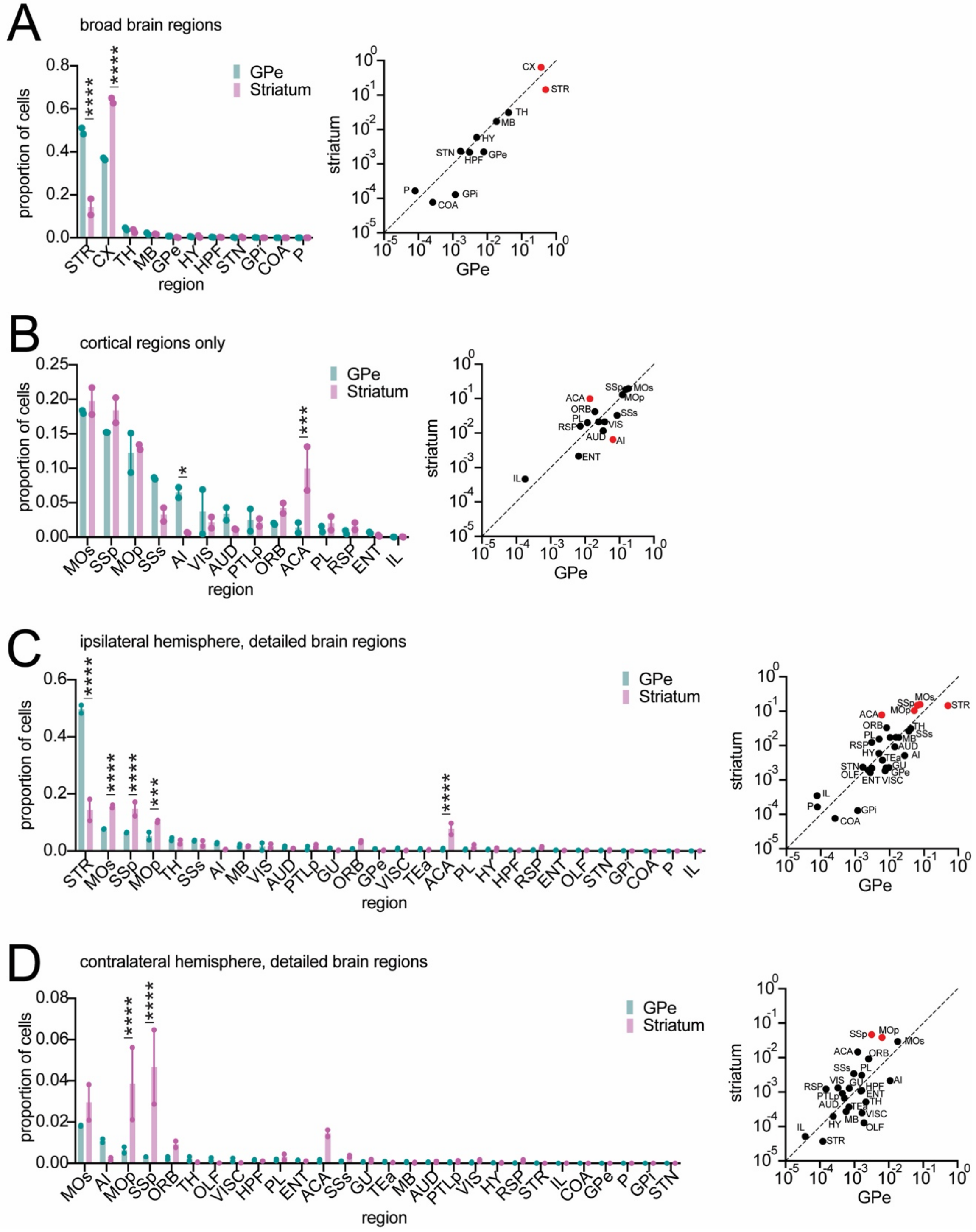
A
broad brain regions
proportion of cells
GPe
Striatum
region
STR CX TH MB GPe HY HPF STN GPi COA P
striatum
GPe
CX STR TH MB HY STN HPF GPe P GPi COA
B
cortical regions only
MOs SSp MOp SSs AI VIS AUD PTLp ORB ACA PL RSP ENT IL
C
ipsilateral hemisphere, detailed brain regions
STR MOs SSp MOp TH SSs AI MB VIS AUD PTLp GU ORB GPe VISC TEa ACA PL HY HPF RSP ENT OLF STN GPi COA P IL
D
contralateral hemisphere, detailed brain regions
MOs AI MOp SSp ORB TH OLF VISC HPF PL ENT ACA SSs GU TEa MB AUD PTLp VIS HY RSP STR IL COA GPe P GPi STN

Figure S8.

Analysis of rabies tracing of corticopallidal versus corticostriatal inputs

A, Proportion of ipsilateral cells innervating the GPe vs striatum, stratified by broad brain regions (n=2 mice), same data as in Figure 4d. There was a significant difference between the GPe and striatum for cortical input ($p<0.0001$) and striatal input ($p<0.0001$), after multiple comparison correction (two-way ANOVA with Šídák's multiple comparison test).

B, Proportion of all ipsilateral cortical cells innervating the GPe vs striatum, stratified by cortical region (n=2 mice). There was a significant difference between the GPe and striatum for agranular insular area ($p=0.0395$) and anterior cingulate area ($p=0.0006$), after multiple comparison correction (two-way ANOVA with Šídák's multiple comparison test).

C, Proportion of all ipsilateral cells innervating the GPe vs striatum, stratified by detailed brain region (n=2 mice). There was a significant difference between the GPe and striatum for the primary somatosensory cortex, secondary somatosensory cortex, secondary motor cortex, anterior cingulate cortex ($p<0.0001$) and primary motor cortex ($p = 0.0007$), after multiple comparison correction (two-way ANOVA with Šídák's multiple comparison test).

D, Proportion of all contralateral cells innervating the GPe vs striatum, stratified by detailed brain region (n=2 mice). There was a significant difference between the GPe and striatum for the primary motor and somatosensory cortices ($p<0.0001$) after multiple comparison correction (two-way ANOVA with Šídák's multiple comparison test).

Key, STR: striatum, MOp: primary motor areas, MOs: secondary motor areas, SSp: primary somatosensory areas, SSs: secondary somatosensory areas, ACA: anterior cingulate areas, ORB: orbital areas, PL: prelimbic areas, IL: infralimbic areas, AI: anterior insular area, GU: gustatory area, VISC: visceral area, AUD: auditory areas, VIS: visual areas, TEa: temporal association areas, PTLp: posterior parietal association areas, RSP: retrosplenial areas, ENT: entorhinal areas, OLF: olfactory areas, HPF: hippocampal formation, STR: striatum, GPe: globus pallidus externa, GPi: globus pallidus interna, TH: thalamus, HY: hypothalamus, STN: subthalamic nucleus, COA: cortical amygdala area, P: pons.

Fig. S9

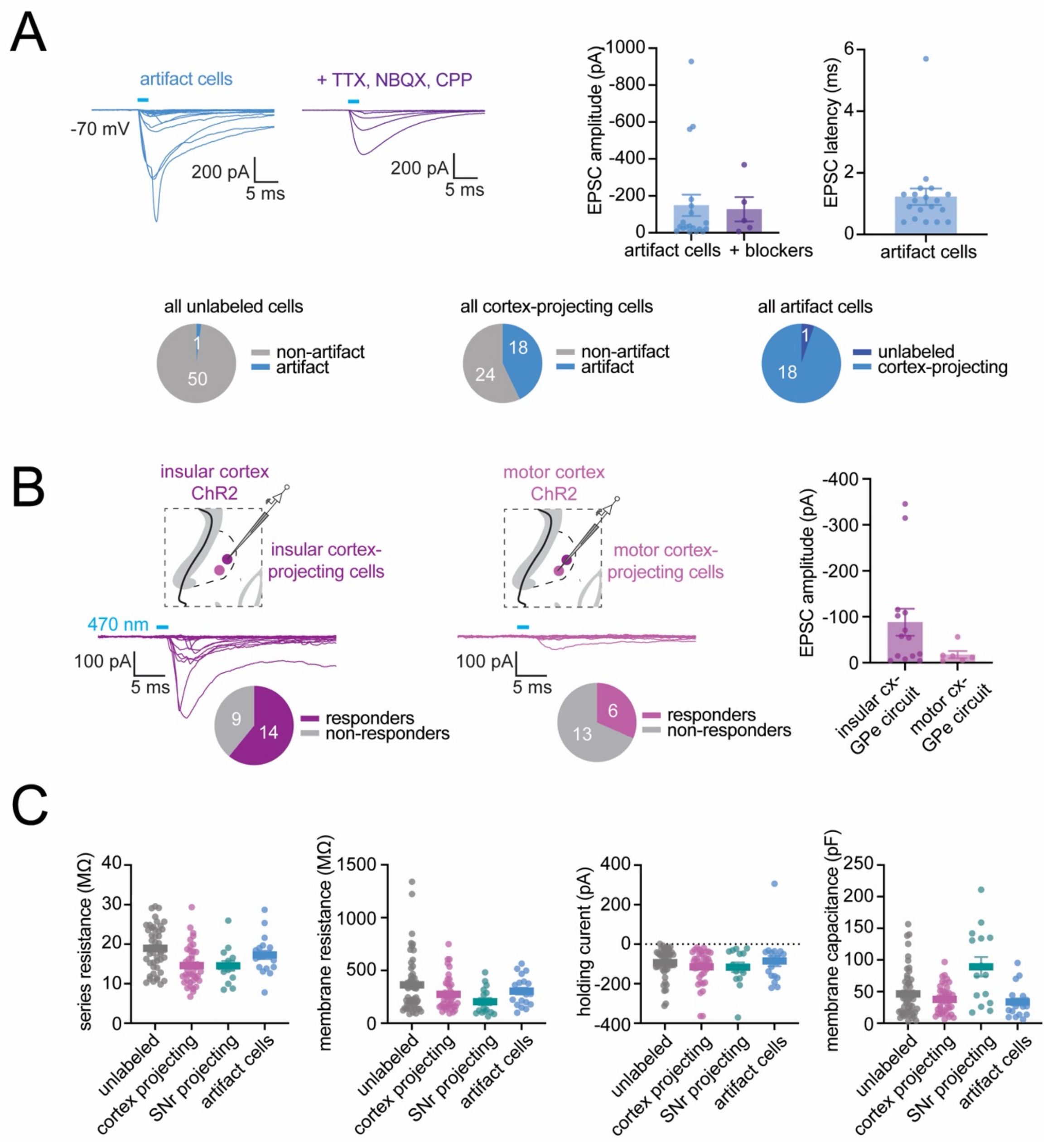
A
artifact cells
+ TTX, NBQX, CPP
-70 mV
200 pA
5 ms
EPSC amplitude (pA)
artifact cells
+ blockers
EPSC latency (ms)
all unlabeled cells
non-artifact
artifact
all cortex-projecting cells
all artifact cells
unlabeled
cortex-projecting
B
insular cortex ChR2
insular cortex-projecting cells
motor cortex ChR2
motor cortex-projecting cells
470 nm
100 pA
responders
non-responders
insular cx-GPe circuit
motor cx-GPe circuit
C
series resistance (MΩ)
membrane resistance (MΩ)
holding current (pA)
membrane capacitance (pF)
unlabeled
cortex projecting
SNr projecting
artifact cells

Figure S9.

Electrophysiological characterization of cortical inputs to GPe in wild-type mice.

A, Large rapid-onset depolarizing currents were seen in 18 cortex-projecting cells (out of a total of 42 cortex-projecting cells) and in 1 unlabeled cell (out of a total of 51 unlabeled cells). We termed these currents "artifact currents" (or photocurrents) likely related to retrograde ChR2 expression in pallidocortical neurons. Artifact currents were not suppressed by TTX and glutamate receptor blockers. *right*, Summary of EPSC amplitude and latency for all neurons with "artifact" currents. Mean and SEM are shown.

B, Synaptic currents evoked by ChR2 stimulation of axons (in the absence of TTX or 4AP) from *left,* insular cortex, recording in insular cortex-projecting GPe neurons (n=23 cells, 9 mice) and *middle,* motor cortex, recording in motor cortex-projecting GPe neurons (n=19 cells, 6 mice). The proportions of responding vs non-responding cells are shown below the traces. *right,* Summary of EPSC amplitude for all responding neurons. Mean and SEM are shown. No significant difference between groups (Mann Whitney test U=21, p=0.0663).

C, Quality metrics and intrinsic properties of all neurons included in Figure 4 and Supp. Fig. 7. Mean and SEM are shown.

Fig. S10

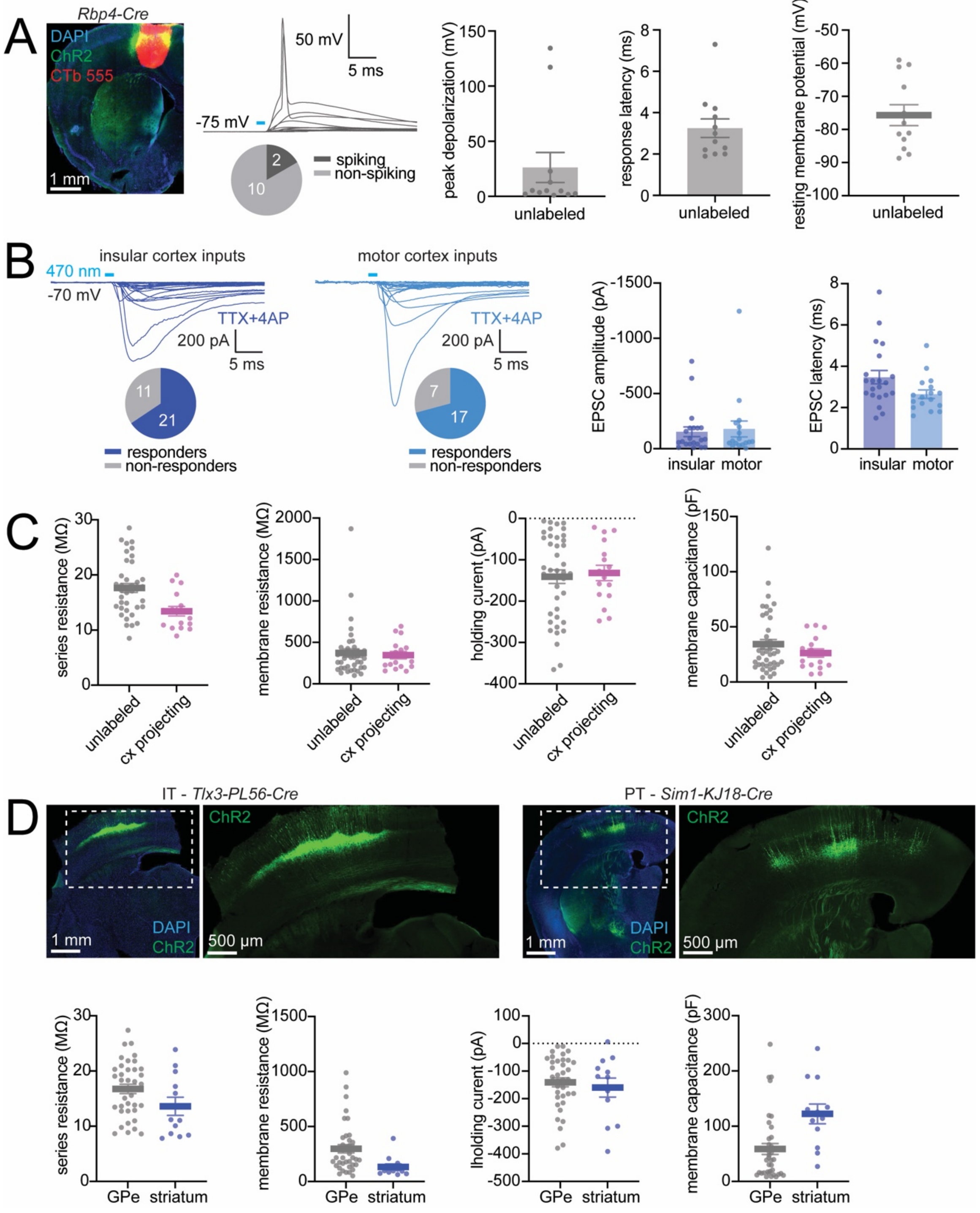

A
Rbp4-Cre
DAPI
ChR2
CTb 555
1 mm
50 mV
5 ms
-75 mV
2
10
spiking
non-spiking
peak depolarization (mV)
unlabeled
response latency (ms)
unlabeled
resting membrane potential (mV)
unlabeled
B
insular cortex inputs
470 nm
-70 mV
TTX+4AP
200 pA
5 ms
11
21
responders
non-responders
motor cortex inputs
TTX+4AP
200 pA
5 ms
7
17
responders
non-responders
EPSC amplitude (pA)
insular
motor
EPSC latency (ms)
insular
motor
C
series resistance (MΩ)
membrane resistance (MΩ)
holding curent (pA)
membrane capacitance (pF)
unlabeled
cx projecting
D
IT - Tlx3-PL56-Cre
PT - Sim1-KJ18-Cre
DAPI
ChR2
1 mm
500 µm
series resistance (MΩ)
membrane resistance (MΩ)
holding curent (pA)
membrane capacitance (pF)
GPe
striatum

Figure S10.

Current clamp responses of GPe neurons to *Rbp4*+ cortical neuron input.

A, *left,* Example image of motor cortex ChR2 and CTb 555 expression in *Rbp4-Cre* mouse. *middle,* Unlabeled GPe neuron current clamp responses to ChR2 stimulation of Rbp4+ cortical axons. *right,* Peak depolarization, response latency and resting membrane potential of all recorded neurons are shown (mean and SEM are shown, n=12 cells, 6 mice).

B, Synaptic currents evoked by ChR2 stimulation of Rbp4+ axons from *left*, insular cortex (n=32 cells, 7 mice) and *middle,* motor cortex (n=24 cells, 5 mice) to all GPe neuron types (in the presence of TTX and 4AP). *Right,* Summary of EPSC amplitude and onset latency for all responding neurons. Mean and SEM are shown.

C, Quality metrics and intrinsic properties of all neurons from *Rbp4-Cre* experiments (Fig. 5c-e), mean and SEM are shown. Unlabeled neurons: n=40 cells, 11 mice, cortex-projecting neurons: n=16 cells, 8 mice.

D, Example images of ChR2 expression in *Tlx3-PL56-Cre* (IT neurons, with cross-callosal intratelencephalic fibers) or *Sim1-KJ18-Cre* (PT neurons, with descending pyramidal tract fibers).

E, Quality metrics and intrinsic properties of all neurons from *Tlx3-PL56-Cre* and *Sim1-KJ18-Cre* experiments (Fig. 5f,g)**,** mean and SEM are shown. GPe neurons: n=37 cells, 4 mice; striatal neurons: n=12 neurons, 4 mice.

Fig. S11

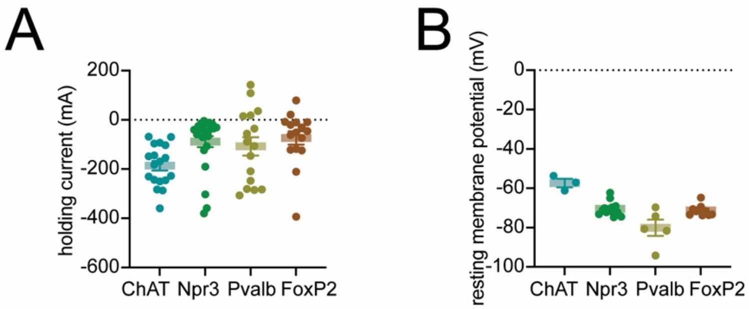

Figure S11.

Intrinsic properties of different GPe cell types.

A, Mean holding current for all neurons shown in Fig. 6B and 6D, mean and SEM are shown. ChAT+ n=18 cells, 2 mice, Npr3+ n=24 cells, 5 mice, Pvalb+ n=16 cells, 3 mice, FoxP2+ n=16 cells, 4 mice.

B, Resting membrane potential of neurons (that were not spontaneously spiking at baseline) shown in Fig. 6C and 6D, mean and SEM are shown. ChAT+ n=3 cells, 2 mice. Npr3+ n=13 cells, 5 mice, Pvalb+ n=5 cells, 2 mice, FoxP2+ n=10 cells, 4 mice.

Fig. S12

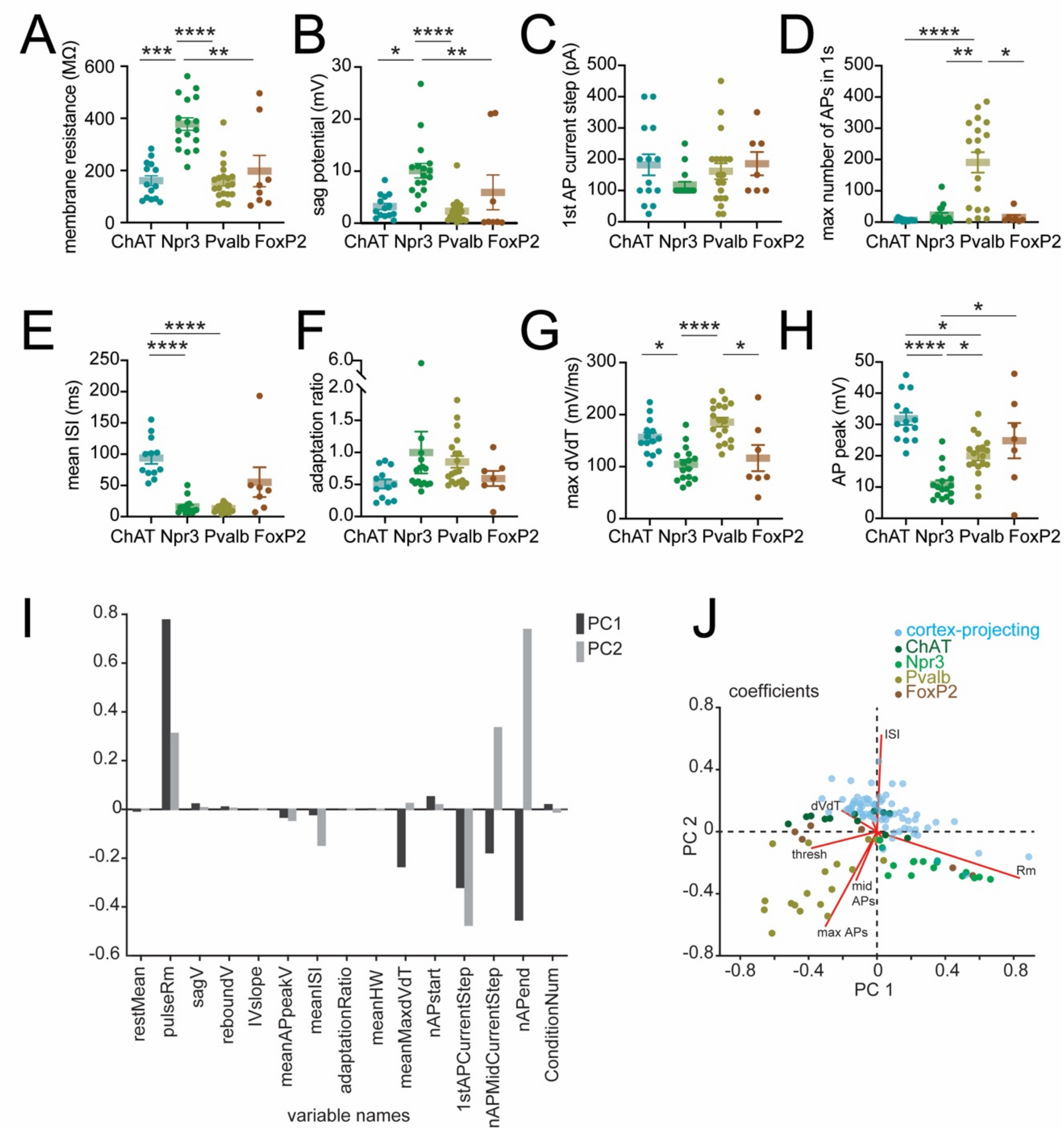

A
membrane resistance (MΩ)
ChAT Npr3 Pvalb FoxP2
B
sag potential (mV)
ChAT Npr3 Pvalb FoxP2
C
1st AP current step (pA)
ChAT Npr3 Pvalb FoxP2
D
max number of APs in 1s
ChAT Npr3 Pvalb FoxP2
E
mean ISI (ms)
ChAT Npr3 Pvalb FoxP2
F
adaptation ratio
ChAT Npr3 Pvalb FoxP2
G
max dVdT (mV/ms)
ChAT Npr3 Pvalb FoxP2
H
AP peak (mV)
ChAT Npr3 Pvalb FoxP2
I
PC1
PC2
restMean
pulseRm
sagV
reboundV
IVslope
meanAPpeakV
meanISI
adaptationRatio
meanHW
meanMaxdVdT
nAPstart
1stAPCurrentStep
nAPMidCurrentStep
nAPend
ConditionNum
variable names
J
cortex-projecting
ChAT
Npr3
Pvalb
FoxP2
coefficients
ISI
dVdT
thresh
mid APs
max APs
Rm
PC 2
PC 1

Figure S12.

Intrinsic properties and principal components analysis for anatomically-labeled pallidocortical neurons and molecularly-labeled GPe neurons

A-H. Quantification of intrinsic properties for different GPe cell types. Mean and SEM are shown. ChAT+ n=14 cells, 2 mice, Npr3 n=17 cells, 5 mice, Pvalb+ n=20 cells, 2 mice, FoxP2+ n= 9 cells, 4 mice. There was no significant difference in the current step required to generate a first action potential (1st AP current step), or mean action potential waveform acceleration (mean max dVdT), after multiple comparison correction. There was a significant difference between groups for the following features: membrane resistance (Kruskal-Wallis test: K-W=27.47, p<0.0001, significant results after multiple comparisons correction: ChAT+ vs Npr3+ p=0.0002, Pvalb+ vs Npr3+ p<0.0001, FoxP2+ vs Npr3+ p=0.0036); sag potential (Kruskal-Wallis test: K-W=23.76, p<0.0001, ChAT+ vs Npr3+ p=0.0104, Pvalb+ vs Npr3+ p<0.0001, FoxP2+ vs Npr3+ p=0.0033); max firing frequency (max number of APs in 1 s) (K-W=24.05, p < 0.0001, ChAT+ vs Npr3+ p<0.0001, Pvalb+ vs Npr3+ p=0.0040, Pvalb+ vs FoxP2+ p=0.0315); mean inter-spike interval (ISI) (K-W=30, p < 0.0001, ChAT+ vs Pvalb+ p < 0.0001, ChAT+ vs Npr3+ p<0.0001); action potential waveform acceleration (max dVdT) (K-W=24.27, p<0.0001, ChAT+ vs Npr3+ p=0.0223, Pvalb+ vs Npr3+ p<0.0001, Pvalb+ vs FoxP2+ p=0.0269), and peak action potential depolarization (AP peak) (K-W=30.76, p < 0.0001, ChAT+ vs Pvalb+ p=0.0153; ChAT+ vs Npr3+ p<0.0001, Pvalb+ vs Npr3+ p=0.0444, FoxP2+ vs Npr3+ p=0.0192).

I, Bar chart of variable coefficients from PCA from Fig. 6G. The bar chart shows the coefficients of the variables for the first and second principal components. The x-axis lists the variables, and the y-axis indicates the magnitude of their coefficients. The height of each bar represents the strength and direction of each variable's contribution to the principal component; taller bars signify larger coefficients and greater influence. Positive coefficients are shown above the x-axis, while negative coefficients extend below it.

Variable name abbreviations, restMean: resting membrane potential, pulseRm: membrane resistance, sagV: sag potential, reboundV: rebound potential, IVslope: slope of subthreshold current-voltage relationship, meanAPpeakV: mean peak voltage of all action potentials, meanISI: mean interspike interval between all action potentials, adaptationRatio: mean ratio between the first action potential pair and last action potential pair for each current step, meanHW=mean action potential half-width, meanMaxdVdT: mean maximum rate of change of membrane potential during rising phase of action potential (action potential waveform acceleration), nAPstart: number of action potentials

during first current step to elicit an action potential, 1stAPCurrentStep: magnitude of current step that first elicits an action potential, nAPMidCurrentStep: number of action potentials elicited during a current step midway through the current step protocol, nAPend: number of action potentials elicited during the last current step (typically 280-400pA), conditionNum: condition number refers to the cell-type identity.

J, Combined principal components analysis for intrinsic property data from Fig. 2 (anatomically labeled pallidocortical neurons, n=83 cells, 13 mice) and Fig. 6 (molecularly-labeled GPe neurons, n=52 cells, 14 mice). Biplot of the first two principal components (PC1 and PC2) showing variable coefficients and scores. Data points represent the scores for each cell on PC1 and PC2 and are color-coded according to their anatomic or genetic identity (cortex-projecting, ChAT+, Npr3+, Pvalb+, FoxP2+). Red lines indicate the direction and magnitude of variable coefficients, with length proportional to the contribution of each variable to the principal components.

Fig. S13

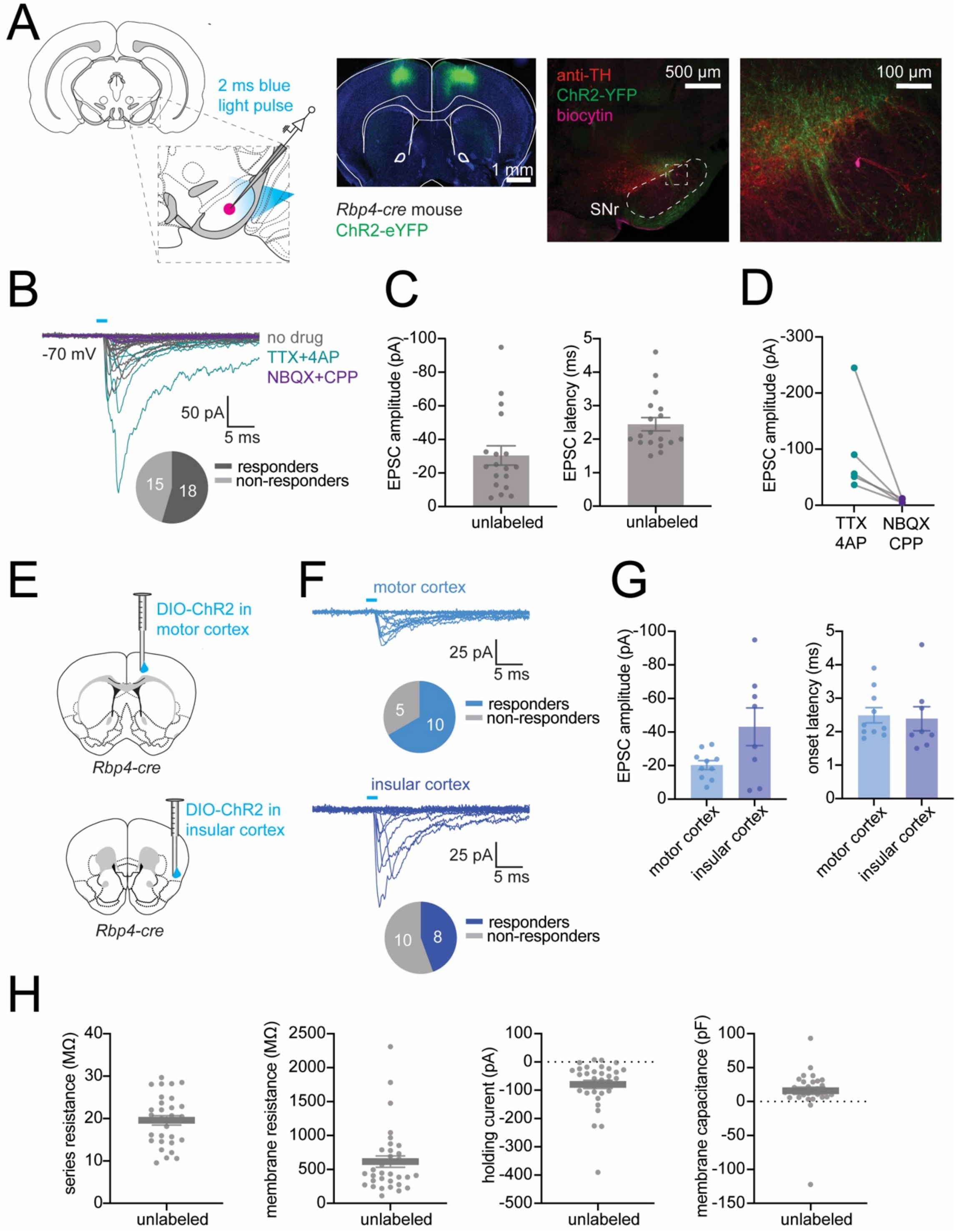
A
2 ms blue light pulse
1 mm
Rbp4-cre mouse
ChR2-eYFP
anti-TH
ChR2-YFP
biocytin
500 µm
SNr
100 µm
B
-70 mV
no drug
TTX+4AP
NBQX+CPP
50 pA
5 ms
15
18
responders
non-responders
C
EPSC amplitude (pA)
unlabeled
EPSC latency (ms)
unlabeled
D
EPSC amplitude (pA)
TTX 4AP
NBQX CPP
E
DIO-ChR2 in motor cortex
Rbp4-cre
DIO-ChR2 in insular cortex
Rbp4-cre
F
motor cortex
25 pA
5 ms
5
10
responders
non-responders
insular cortex
25 pA
5 ms
10
8
responders
non-responders
G
EPSC amplitude (pA)
onset latency (ms)
motor cortex
insular cortex
H
series resistance (MΩ)
membrane resistance (MΩ)
holding curent (pA)
membrane capacitance (pF)
unlabeled

Figure S13.

Cortical inputs to neurons in the substantia nigra pars reticulata (SNr).

A, *left,* Schematic of experimental approach: *Cre*-dependent ChR2 was injected into the cortex of *Rbp4-Cre* mice. Neurons in the SNr were patched during stimulation of ChR2 with 2 ms pulses of 470 nm light. *right,* Example histological images of the cortical injection site and patched neurons in the SNr are shown. Anti-tyrosine hydroxylase (TH) stain is used to indicate the location of dopamine neurons in the substantia nigra pars compacta (SNc) which is dorsal to the SNr.

B, Voltage clamp responses of SNr neurons to stimulation of cortical axons in the SNr in the absence of any pharmacologic agents, and in the presence of TTX+4AP (to isolate monosynaptic inputs) and glutamatergic receptor blockers (NBQX and CPP) to block glutamatergic input. Proportion of neurons responding to cortical input is shown below the traces.

C, Quantification of EPSC amplitude and EPSC latency in all responding cells. Mean and SEM are shown, n=18 cells, 5 mice.

D, EPSC amplitude in the absence and presence of glutamatergic blockers, n=5 neuron pairs from 4 mice (Wilcoxin matched-pairs signed rank test: W=15.00, p=0.0625).

E, ChR2 was injected either into motor cortex or insular cortex. Excitatory synaptic inputs were recorded in SNr neurons in response to stimulation of ChR2-expressing cortical axons.

F, Voltage clamp responses to blue light stimulation of motor cortex and insular cortex inputs. Proportion of responding neurons are shown below the traces.

G, Quantification of EPSC amplitude and latency. Mean and SEM are shown. Motor cortex n=15 cells, 2 mice, insular cortex n=18 cells, 3 mice.

H, Summary of intrinsic properties of all SNr neurons.

Supplementary Methods

Intracranial injections

Mice were anesthetized with 3% isoflurane and maintained under anesthesia during surgery with 1.5% isoflurane and 80% oxygen. Using a stereotactic frame (David Kopf Instruments), the skull was exposed under aseptic conditions, a small craniotomy (around 300 μm) was drilled and cholera toxin b (CTb) or virus (non-pseudotyped rabies virus or AAV) (*SI Appendix,* Table S1, Materials and Resources) was injected into the assigned brain region(s) at the coordinates listed in *SI Appendix,* Table S2, Stereotactic Injections). All coordinates are in mm and are given relative to bregma (AP and ML) and skull (DV) as defined in The Paxinos and Franklin Mouse Brain Atlas[2]. Injection volumes and expression times are listed in *SI Appendix,* Table S2, Stereotactic Injections).

Injections were performed as previously described[3–5]. A pulled glass pipette was briefly lowered 200 μm past the target depth, then retracted and held in place at the target depth for 2 min prior to injection, then and CTb or viruses were infused at a rate of 50 nL min^{-1} using a syringe pump (Harvard Apparatus, 883015). Pipettes were slowly withdrawn at least 5 min after the end of the infusion. After injections, the wound was sutured. After surgery, mice were placed in a recovery cage with a heating pad until their activity was recovered, before being returned to their home cage. Mice were given pre- and post-operative oral carprofen (CPF, 5 mg per kg per day) analgesia and monitored daily for at least four days post-surgery. For retrograde tracer injections (rabies virus, CTb), 5-7 days were allowed for expression before experiments were performed. For *in vitro* electrophysiology experiments, ChR2 virus was allowed to express for 3-8 weeks before experiments were performed (mean expression time = 4.4 weeks, std = 1.2 weeks).

Transsynaptic rabies virus tracing injections

For transsynaptic retrograde tracing, injections were performed as described above. *ChAT-IRES-Cre* and *Npr3-IRES-Cre* mice (2-5 months) were injected with *Cre*-dependent TVA helper virus (AAV8-EF1a-FLEX-GT) into the GPe, which was allowed to express for 2 weeks prior to injection of a pseudotyped rabies virus (CVS-N2c-dG-mCherry (EnvA)) into the motor or insular cortex of the same mice. Two control experiments were performed: 1) AAV8-EF1a-FLEX-GT was injected into the GPe of a WT mouse to confirm

that TVA expression was dependent on *Cre*-expression (Supp. Fig. 5c) 2) CVS-N2c-dG-mCherry (EnvA)) was injected into the cortex of a WT mouse (without prior TVA injection) to confirm that mCherry expression was dependent on TVA expression.

Histology and immunohistochemistry

Histology for anatomical studies

Mice were deeply anesthetized with isoflurane and transcardially perfused with 5–10 ml chilled phosphate buffered saline (PBS), followed by 10–15 ml chilled 4% paraformaldehyde in PBS. Brains were dissected out and post-fixed overnight at 4°C, and transferred either to 0.1M PBS or incubated in a storing/cryoprotectant solution of 30% sucrose and 0.05% sodium azide in PBS. Brains were allowed to equilibrate for at least two days. Brains stored in PBS were sectioned into slices at 50µm thickness on a vibratome (Leica VT1000 S). Brains stored in 30% sucrose cryoprotectant solution were sectioned on a freezing microtome (Leica Biosystems, SM2010 R) before being transferred to PBS. Slices were then mounted and coverslipped with ProLong Diamond Antifade Mountant with DAPI (Thermo Fisher Scientific). Slides were imaged with an Olympus VS120 or VS200 slide scanning microscope.

Histology and biocytin staining for electrophysiological slices

300µm thick brain slices used in electrophysiology experiments were fixed for 24 hours in chilled 4% PFA in a 24 well plate after recordings were complete. Slices were then incubated in a storing/cryoprotectant solution of 30% sucrose and 0.05% sodium azide in PBS for at least 2 days (or until they sank). Slices were then mounted onto a block of Tissue-Tek® O.C.T. Compound in a cryostat microtome (Leica Biosystems, CM1950) and re-sliced into 50 µm thick sections. The sections were then transferred to PBS for biocytin staining. For staining biocytin-filled cells with streptavidin, slices were rinsed 3 x 10 min in PBS. On the 3rd wash, slices were blocked in PBS + 0.2% TritonX + 3% normal goat serum (NGS) for two hours at room temperature. After two hours of blocking, the slices were incubated in 1:1000 dilution of Streptavidin Alexa Fluor™ 647 Conjugate, 2mg/mL (Invitrogen catalog no. S32357) in blocking solution for two hours. The sections were then

rinsed 3 x 10 min in PBS, before being mounted, coverslipped and imaged as described above.

Analysis

Anatomical analyses

Sagittal images of retrograde CTb tracing experiments were manually aligned to sagittal images from the Allen Brain Atlas to represent "lateral" (ML = 2.725mm), "intermediate" (ML = 2.35mm), and "medial" (ML = 1.95mm) sagittal planes. Zoomed sections of GPe for each Allen Brain Atlas Image were then used to manually annotate the location of cells expressing different CTb fluorophores in GPe, corresponding to different cortical projection targets. Co-labeling of cells with two or more CTb fluorophores was also annotated. The number of cells projecting to each cortical projection target (motor, sensory, insular, prefrontal, or colabeled cells) was quantified for each sagittal section.

mRNA FISH analysis

The rabies channel images were used to create a mask of rabies expression using Otsu thresholding or a custom machine-learning algorithm in MATLAB, to automatically segment rabies-infected cells. Slices were discarded if excessive background fluorescence caused incorrect cell segmentation. RNAscope puncta quantification was performed using a custom machine learning algorithm in MATLAB (developed by the HMS Neuroimaging Facility). Puncta counts were exported as csv files for further analysis and data visualization using custom scripts in MATLAB and using GraphPad. Bootstrap analyses of the Spearman correlation coefficient for puncta counts for two different mRNA markers within individual cells was performed using 1000 bootstrap repetitions. The bootstrap analysis was repeated for the same datasets after random shuffling of the data. If the actual data bootstrap distribution was separated from the shuffled distribution by more than the mean of the Spearman correlation coefficient (r) +/- two standard deviations from the mean, we considered the Spearman correlation between the markers statistically significant. Poisson based minimum error thresholding was used to classify cells as "expressing" or "non-expressing" for a given mRNA marker[6,7].

NeuroInfo whole brain cell quantification pipeline

Coronal images were imported to NeuroInfo software (MBF Bioscience, Williston, VT) for alignment and registration to the Allen Mouse Brain Atlas. All images were inspected and adjusted manually as necessary for correct alignment. Cells were detected automatically using the "Cell Detection Pipeline", and all images were inspected carefully for accurate detection and edited manually as necessary to remove obvious false positive detections. The cell detection data was extracted and analyzed further using custom MATLAB scripts.

Electrophysiological data quality control and analysis

Electrophysiological recording traces were analyzed using custom MATLAB scripts. The resting membrane potential (RMP) is only reported in neurons that were not spontaneously spiking at baseline. For experiments in Fig. 2, 4, 5 and 6, and Supp. Fig. 3, 9, 10, 11, 12 and 13 in which we analyzed intrinsic properties (current clamp) and evoked cortical inputs (voltage clamp and current clamp), we set the resting membrane potential to -70 mV. In current clamp, this cannot be done exactly, therefore we report the range of RMPs that we attained. Cells were excluded from further analysis if the RMP was more hyperpolarized than -90mV or more depolarized than -50 mV, or the standard deviation of the baseline membrane potential was greater than 5 mV. For the current clamp experiment in Fig. 6e in which cells were spontaneously active, we did not set the membrane potential. We used these recordings to examine input-evoked changes in spiking timing. In voltage clamp, the current necessary to hold the membrane potential at -70 mV is reported as the holding current. Cells were excluded from further analysis if the holding current (Ih) was more negative than -400 pA, series resistance was greater than 40MΩ or the series resistance varied by more than 20% over the course of all traces for that cell. Cells were classified as "responders" to optogenetic stimulation if the change in membrane current after the light pulse was greater than 4 times the standard deviation of the baseline membrane current prior to the onset of the light pulse.

Table S1.

Materials and Resources

**Reagent type**	**Designation**	**Source or reference**	**Identifiers**	**Additional information**
Strain, strain background (*M. musculus)*	Wild-type, C57BL6/J	Jackson Labs	Stock # 00644	
Strain, strain background (*M. musculus)*	*Drd2-eGFP*	GENSAT	MGI # 3843608	*Tg(Drd2-EGFP)S118Gsat*
Strain, strain background (*M. musculus)*	*Rbp4-Cre-KL100*	GENSAT	MGI # 4367067	*Tg(Rbp4-cre)KL100Gsat*
Strain, strain background (*M. musculus)*	*Tlx3-PL56-Cre*	GENSAT	MGI # 5311700	*Tg(Tlx3-cre)PL56Gsat*
Strain, strain background (*M. musculus)*	*Sim1-KJ18-Cre*	GENSAT	MGI # 4367070	*Tg(Sim1-cre)KJ18Gsat*
Strain, strain background (*M. musculus)*	*PV-IRES-Cre*	Jackson Labs	Stock # 017320	*B6.129P2-* $Pvalb^{tm1(cre)Arbr}$*/J*
Strain, strain background (*M. musculus)*	*Ai14* (tdTomato *Cre*-dependent reporter line)	Jackson Labs	Stock # 007914	*B6.Cg-* $Gt(ROSA)26Sor^{tm14(CAG-tdTomato)Hze}$*/J*
Strain, strain	*PV-IRES-Cre* x *Ai14*			

background (*M. musculus)*				
Strain, strain background (*M. musculus)*	*ChAT-IRES-Cre*	Jackson Labs	Stock # 006410	*B6;129S6-Chat$^{tm2(cre)Lowl}$/J*
Strain, strain background (*M. musculus)*	*ChAT-IRES-Cre* x *Ai14*			
Strain, strain background (*M. musculus)*	*FoxP2-IRES-Cre*	Jackson Labs	Stock # 030541	*B6.Cg-Foxp2$^{tm1.(cre)Rpa}$/J*
Strain, strain background (*M. musculus)*	*Npr3-IRES2-Cre-*	Jackson Labs	Stock # 031333	*B6.Cg-Npr3$^{tm1.1(cre)Hze}$/J*
Genetic reagent (rabies virus)	B19G-SADΔG-tdTomato; RbV-tdTomato	Generated in-house (see Huang et al., 2019[4])		2.93 x 10^10 IU/mL
Genetic reagent (rabies virus)	B19G-SADdG-H2B:EGFP; RbV-H2B:GFP	Generated in-house (see Huang et al., 2019[4]), Janelia virus core		10^9-10^10 IU/mL
Genetic reagent (rabies virus)	CVS-N2c-dG-tdTom (no envelope)	Janelia vírus core		1.92 x 10^8 IU/mL
Genetic reagent (rabies virus)	CVS-N2c-dG-tdTom (no envelope)	Janelia vírus core		5.32 x 10^8 IU/mL
Genetic reagent (rabies vírus)	CVS-N2c-dG-mCherry (EnvA)	Janelia vírus core		1.4 X 10^10 IU/mL

Genetic reagent (AAV)	AAV8-EF1a-FLEX-TVA-GFP	Salk Institute		3.27 X 10^13 gc/mL
Genetic reagent (AAV)	AAV9-EF1a-DIO-hChR2(H134R)-eYFP	Addgene	Stock # 20298-AAV9	1.8 × 10^13 gc/mL
Protein conjugate	Cholera Toxin Subunit B (Recombinant), Alexa Fluor™ 488 Conjugate	ThermoFisher	# C34775	4µg/µL
Protein conjugate	Cholera Toxin Subunit B (Recombinant), Alexa Fluor™ 555 Conjugate	ThermoFisher	# C34776	4µg/µL
Protein conjugate	Cholera Toxin Subunit B (Recombinant), Alexa Fluor™ 647 Conjugate	ThermoFisher	# C34778	4µg/µL
Protein conjugate	Streptavidin, Alexa Fluor™ 647 Conjugate	ThermoFisher	# S32357	2mg/mL, 1:1000 dilution
Primary antibody	Rabbit RFP antibody	Rockland	600-401-379	1:1000 dilution
Primary antibody	Chicken GFP antibody	ThermoFisher	A10262	1:1500, in glycerol 1:2
Primary antibody	Rabbit tyrosine hydroxylase antibody	Pel-Freez Biologicals	# P40101-150	1:1000 dilution
Secondary antibody	Goat anti-rabbit 555	ThermoFisher	A-21429	1:1000 dilution
Secondary antibody	Goat anti-chicken 488	ThermoFisher	A-11039	1:1000 dilution
Commercial assay, kit	RNAscope V1 fluorescent multiplex detection assay reagents	ACDBio	# 320851	
Commercial assay, kit	RNAscope V1 fluorescent multiplex detection assay, protease	ACDBio	# 322340	
Commercial assay, kit	RNAscope V1 fluorescent multiplex	ACDBio	# 408731<br># 319191<br># 524021<br># 434721	Mm-ChAT<br>Mm-Slc32a1<br>Mm-Fibcd1<br>Mm-Nkx2.1

	detection assay, probes		# 468851<br># 502991<br># 461908<br># 406501<br># 456781	Mm-Npas1<br>Mm-Npr3<br>Mm-Drd1<br>Mm-Drd2<br>V-RABV-gp1
Chemical compound	ProLong™ Gold Antifade Mountant	ThermoFisher	# P36934	
Chemical compound	ProLong™ Diamond Antifade Mountant with DAPI	ThermoFisher	# P36971	
Chemical compound	Biocytin	Sigma	# B4261	1mg/mL in internal solution
Chemical compound, drug	Gabazine	Tocris	# 1262	10 μM
Chemical compound, drug	TTX	Tocris	# 1069	10 μM
Chemical compound, drug	4AP	Sigma	# A78403	400 μM
Chemical compound, drug	NBQX	Tocris	# 0373	10 μM
Chemical compound, drug	CPP	Sigma	# C104	10 μM

Table S2.

Stereotactic Injections

**Brain region**	**Stereotactic coordinate (Paxinos and Franklin[2]) (mm)**	Injection volume
Motor cortex	Injection 1: AP +1.1, ML +/-1.3, DV 1.1, 1.35<br>Injection 2: AP +0.7, ML +/-1.2, DV 1.1, 1.35	150 nL at each depth
Sensory cortex	Injection 1: AP 0, ML +/-2.75, DV 1.5, 1.7<br>Injection 2: AP -1, ML +/-3.25, DV 1.5, 1.7	150 nL at each depth
Insular cortex	Injection 1: AP +1.5, ML +/-3.3, DV 3.4, 3.75<br>Injection 2: AP +1.1, ML +/-3.4, DV 3.2, 3.4	150 nL at each depth
Prefrontal cortex	Injection 1: AP 2.1, ML +/-0.3, DV 1.2, 1.7<br>Injection 2: AP 2.3, ML +/-0.2, DV 1.3, 2.0	150 nL at each depth
GPe	Figure 3h: AP: -0.800, ML: +/-2.25, DV: -4.000<br>Figure 4a-d: AP -0.6, ML +/-2.38, DV -4.1	Figure 3h: 150 nL<br>Figure 4a: 75 nL
Striatum	Figure 4a-d: AP +0.1, ML +/-2.0, DV -3.0	300 nL
SNr	Figure 4g: AP -3.2, ML +/-1.5, DV -4.5	300 nL

Table S3.

Statistical Tests

**Figure panel**	**Statistical test**	**Test value**	**p-value**	**Details (for significant results)**
Fig. 2c Sig. results	Kruskal-Wallis test<br>-sagV	K-W=9.102	p=0.028	Dunn's MC test<br>MCx vs InsCx p = 0.0180
Fig. 2d Sig. results	Kruskal-Wallis test<br>-meanISI	K-W=16.65	p=0.0008	Dunn's MC test<br>MCx vs InsCx p = 0.0107<br>SCx vs InsCx p = 0.0014
Fig. 3b	Spearman correlation	r=-0.3019	p<0.0001	*Slc32a1* vs *Chat*
Fig. 3d	Spearman correlation	r=0.6651<br>r=-0.1505	p<0.0001<br>p=0.1975	*Nkx2-1 vs Chat*<br>*Nkx2-1 vs Slc32a1*
Fig. 3e	Spearman correlation	r=-0.05301<br>r=0.4955	p=0.507<br>p<0.0001	*Npr3 vs Chat*<br>*Npr3 vs Slc32a1*
Fig. 4b	Two-tailed Mann Whitney U test	U=0	P=0.333	
Fig. 4c	Two-way ANOVA<br>-brain region<br>-GPe-striatum<br>-interaction term	 F(10,22)=657.4<br>F(1,22)=5.110<br>F(10,22)=114.1	 p<0.0001<br>=0.0340<br>p<0.0001	Šídák's MC test<br>striatum p<0.0001<br>cortex p<0.0001
Fig. 4d	Two-way ANOVA<br>-layer<br>-GPe-striatum<br>-interaction term	 F(5,12)=1898<br>F(1,12) = 0.000<br>F(5,12)=16.74	 p<0.0001<br>p>0.9999<br>p<0.0001	Šídák's MC test<br>layer 2-3 p=0.0001<br>layer 5 p=0.0014
Fig. 4h	Kruskal-Wallis test<br>-EPSC amplitude<br>-latency	 K-W=3.914<br>K-W=0.6003	 p=0.1413<br>p=0.7407	Dunn's MC test<br>ns after MC correction<br>ns after MC correction
Fig. 4j	Two-tailed Mann Whitney U test<br>-EPSC amplitude<br>-latency	 U=312<br>U=390	 p=0.0139<br>p=0.1670	
Fig. 5d	Wilcoxin Signed Rank test<br>-unlabeled<br>-cortex-projecting	 W=45<br>W=21	 p=0.0039<br>p=0.0312	
Fig. 5e	Two-tailed Mann Whitney U test<br>-EPSC amplitude	U = 0	p=0.0303	
Fig. 5f	Two-tailed Mann Whitney U test<br>-GPe amplitude<br>-GPe latency	 U=9<br>U=18.50<br>U=5	 p=0.0289<br>p=0.2953<br>p=0.2667	

	-striatum amplitude<br>-striatum latency	U=9	p=0.8333	
Fig. 6d	Kruskal-Wallis test<br>-EPSC amplitude<br>-peak depolarization<br>-latency	K-W=7.653<br>K-W=1.467<br>K-W=5.840	p=0.0538<br>p=0.6899<br>p=0.1197	Dunn's MC test<br>PV vs Npr3 p = 0.0397<br>ns after MC correction<br>ns after MC correction
Fig. 6e	Two-tailed paired t-test<br>-ChAT neurons<br>-Pvalb neurons	t=1.084 (df=9)<br>t=2.910 (df=4)	p=0.3065<br>p=0.0437	
Supp. Fig. 4b	Spearman correlation	r=0.205<br>r=0.5989	p=0.0718<br>p<0.0001	*Npas1 vs Chat*<br>*Npas1 vs Slc32a1*
Supp. Fig. 4c	Spearman correlation	r=0.4055<br>r=0.5653	p<0.0001<br>p<0.0001	*Fibcd1 vs Chat*<br>*Fibcd1 vs Slc32a1*
Supp. Fig. 4d	Spearman correlation	r=0.40	p<0.0001	*Drd1 vs Drd2*
Supp. Fig. 8a	Two-way ANOVA<br>-brain region<br>-GPe-striatum<br>-interaction term	F(10,22)=657.4<br>F(1,22)=5.110<br>F(10,22)=114.1	p<0.0001<br>=0.0340<br>p<0.0001	Šídák's MC test<br>striatum p<0.0001<br>cortex p<0.0001
Supp. Fig. 8b	Two-way ANOVA<br>-cortical region<br>-GPe-striatum<br>-interaction term	F(13,28)=47.08<br>F(1,28)=0.1490<br>F(13,28)=3.934	p<0.0012<br>p=0.7025<br>p<0.0001	Šídák's MC test<br>AI p=0.0395<br>ACA p=0.0006
Supp. Fig. 8c	Two-way ANOVA<br>-brain region<br>-GPe-striatum<br>-interaction term	F(27,56)=133.0<br>F(1,56)=2.092<br>F(27,56)=43.67	p<0.0001<br>p=0.1537<br>p<0.0001	Šídák's MC test<br>Str,SSp,Mos,SSs,ACA p<0.0001<br>MOp p=0.0007
Supp. Fig. 8d	Two-way ANOVA<br>-brain region<br>-GPe-striatum<br>-interaction term	F(27,56)=8.362<br>F(1,56)=13.56<br>F(27,56)=4.371	p<0.0001<br>p=0.0005<br>p<0.0001	Šídák's MC test<br>MOp,SSp p<0.0001
Supp. Fig. 9b	Two-tailed Mann Whitney U test<br>-EPSC amplitude	U=21	P=0.0913	
Supp. Fig. 9c	Kruskal-Wallis test<br>-Rs	K-W=16.76	p=0.0008	Dunn's MC test<br>unlabeled vs cortex-projecting p=0.0012;<br>unlabeled vs SNr-projecting p=0.0484
	-Rm	K-W=7.955	p=0.0470	unlabeled vs SNr-projecting p= 0.0435
	-Iholding	K-W=1.899	p=0.5936	ns after MC correction
	-Cm	K-W=11.30	p=0.0102	unlabeled vs SNr-projecting p=0.0406;

				cortex-projecting vs SNr-projecting p=0.0251;<br>SNr-projecting vs artifact p=0.0090
Supp. Fig. 10b	Two-tailed Mann Whitney U test<br>-EPSC amplitude<br>-latency	U=177<br>U-107.5	p=0.9769<br>p=0.0364	
Supp. Fig. 10c	Two-tailed Mann Whitney U test<br>-Rs<br>-Rm<br>-Iholding<br>-Cm	U=150<br>U=307<br>U=314<br>U=281	p=0.0030<br>p=0.8225<br>p=0.9214<br>p=0.4889	
Supp. Fig. 10d	Two-tailed Mann Whitney U test<br>-Rs<br>-Rm<br>-Iholding<br>-Cm	U=141<br>U=95<br>U=207<br>U=85	p=0.0759<br>p=0.0024<br>p=0.7394<br>p=0.0010	
Supp. Fig. 11a	Kruskal-Wallis test<br>-Iholding	K-W=13.86	p=0.0031	Dunn's MC test<br>ChAT vs FoxP2 p=0.0067<br>ChAT vs Npr3 p=0.0076
Supp. Fig. 11b	Kruskal-Wallis test<br>-resting V	K-W=12.24	p=0.0066	Dunn's MC test<br>ChAT vs Pvalb p=0.0032
Supp. Fig. 12a	Kruskal-Wallis test<br>-Rm	K-W=27.47	p<0.0001	Dunn's MC test<br>ChAT vs Npr3 p=0.0002<br>PV vs Npr3 p<0.0001<br>FoxP2 vs Npr3 p=0.0036
Supp. Fig. 12b	-sagV	K-W=23.76	p<0.0001	ChAT vs Npr3 p=0.0104; PV vs Npr3 p<0.0001, FoxP2 vs Npr3 p=0.0033
Supp. Fig. 12c	-currStep1stAP	K-W=3.088	p=0.3783	ns
Supp. Fig. 12d	-max num Aps in 1s	K-W=24.05	p<0.0001	ChAT vs PV p<0.0001<br>PV vs FoxP2 p=0.0315<br>PV vs Npr3 p=0.0040
Supp. Fig. 12e	-meanISI	K-W=30	p<0.0001	ChAT vs PV p<0.0001<br>ChAT vs Npr3 p<0.0001
Supp. Fig. 12f	-adaptation ratio	K-W=7.304	p=0.0628	ns
Supp. Fig. 12g	-max dVdT	K-W=24.27	p<0.0001	ChAT vs Npr3 p=0.0223<br>PV vs FoxP2 p=0.0269<br>PV vs Npr3 p<0.0001

Supp. Fig. 12i	-AP peak	K-W=30.76	p<0.0001	ChAT vs PV p=0.0153; ChAT vs Npr3 p<0.0001; PV vs Npr3 p=0.0444, FoxP2 vs Npr3 p=0.0192
Supp. Fig. 13d	Wilcoxin Signed Rank test	W=15	p=0.0625	
Supp. Fig. 13g	Two-tailed Mann Whitney U test<br>-EPSC amplitude<br>-latency	 <br>U=25<br>U=30	 <br>p=0.2031<br>p=0.4063	

Data was tested for normality with the Shapiro-Wilk test. Datasets that were not normally distributed were analyzed with non-parametric statistical tests.

Supplementary References

1. Allen Brain Mouse Atlas (https://mouse.brain-map.org/).
2. George Paxinos & Franklin, Keith. *Paxinos and Franklin's the Mouse Brain in Stereotaxic Coordinates*. (Amsterdam; Boston: Elsevier Academic Press, 1944).
3. Chantranupong, L. *et al.* Dopamine and glutamate regulate striatal acetylcholine in decision-making. *Nature* **621,** 577–585 (2023).
4. Huang, K. W. *et al.* Molecular and anatomical organization of the dorsal raphe nucleus. *eLife* **8**, e46464 (2019).
5. Melzer, S. *et al.* Bombesin-like peptide recruits disinhibitory cortical circuits and enhances fear memories. *Cell* **184**, 5622-5634.e25 (2021).
6. Fan, Juilun. Notes on Poisson distribution-based minimum error thresholding. **19**, 425–431 (1998).
7. Pal, Nikhil & Pal, Sankar. Image model, poisson distribution and object extraction. *International Journal of Pattern Recognition and Artificial Intelligence* **5**, 459–483 (1991).
